# Spermine and thermospermine synthases emerged multiple times during eukaryote evolution

**DOI:** 10.1016/j.jbc.2025.111028

**Published:** 2025-12-09

**Authors:** Bin Li, Jue Liang, Hamid R. Baniasadi, Margaret A. Phillips, Anthony J. Michael

**Affiliations:** Department of Biochemistry, UT Southwestern Medical Center, Dallas, Texas, USA

**Keywords:** polyamine, spermidine, spermine, thermospermine, evolution, eukaryote, fungi, metazoa, archaeplastida, protist, endosymbiotic, neofunctionalization, gene duplication

## Abstract

The polyamines spermine and thermospermine are differentially distributed throughout eukaryotic phyla. It is unlikely that they were present in the Last Eukaryotic Common Ancestor, thus their biosynthetic enzymes, spermine synthase (SpmSyn) and thermospermine synthase (TspmSyn) emerged during eukaryotic evolution. Herein, we show the different evolutionary mechanisms by which functionally validated SpmSyns and TspmSyns evolved, and their phylogenetic distribution in eukaryotes. Animal lineage SpmSyn was horizontally acquired as a bacterial *S*-adenosylmethionine decarboxylase-SpmSyn fusion protein before the emergence of the single-celled closest relatives of animals, the Choanoflagellata. SpmSyn has been lost from comb jellies, some sponge species, and was lost from most free-living and parasitic worms. Corals encode two SpmSyn homologs, one of which has evolved into a TspmSyn. In fungi, SpmSyn evolved by gene duplication of spermidine synthase and subsequent neofunctionalization early in the budding yeast Saccharomycotina subphylum. Similarly, the plant SpmSyn evolved by gene duplication of spermidine synthase and then neofunctionalization in lycophytes, coincident with the emergence of vascularization. TspmSyn is found throughout plants and green algae, but lost from wild and domesticated barley. It was likely acquired by endosymbiotic gene transfer from the cyanobacterial ancestor of the chloroplast, although the closest homolog of plant TspmSyn is from the Chloroflexota. TspmSyn homologs evolved into SpmSyns in red algae and into spermidine synthase in glaucophyte algae. Chloroflexota-type TspmSyns are found in many protist phyla, often correlated with secondary endosymbiosis of red or green algae, but were acquired by horizontal gene transfer in phyla that have not possessed algal plastids.

The polyamine spermidine (Spd) (Fig. 1) is essential for cell growth and proliferation in eukaryotic cells due to its role as source of the aminobutyl group that is used in the hypusine post-translational modification of translation factor eIF5a (1, 2, 3). Hypusination of eIF5a is the only known essential function of Spd conserved in all eukaryotes. Spd also serves as precursor for biosynthesis of the same mass tetraamine structural isomers spermine (Spm) and thermospermine (Tspm) (Fig. 1). Aminopropylation of the *N*^8^-aminobutyl side of Spd produces Spm, and of the *N*^1^-aminopropyl side produces Tspm (4). The transferred aminopropyl group is derived from decarboxylated *S*-adenosylmethionine, supplied by *S*-adenosylmethionine decarboxylase (AdoMetDC), and the aminopropylation is performed by the aminopropyltransferases (APTs) Spm and Tspm synthases (SpmSyn and TspmSyn). Spd levels are tightly controlled in cells by regulation of biosynthesis, catabolism and transport, due to potential interference of eIF5a function by excess or insufficient levels of Spd. This would suggest that Spd-consuming Spm and Tspm biosynthesis requires separate enzymes, rather than a single APT with relaxed substrate specificity that could produce Spd, Spm and Tspm. In this way, Spd content homeostasis can be dissociated from Spm and Tspm biosynthesis. The physiological roles of Spm and Tspm at the molecular level are much less understood than that of Spd. In humans, genetic defects in SpmSyn activity are associated with severe neurological and developmental problems (5, 6). Acrolein is a toxic metabolite produced by Spm catabolism, and its formation from Spm is associated with tissue damage during brain stroke (7). In the model flowering plant *Arabidopsis thaliana*, Tspm is required for stem elongation (8), whereas Spm is required for resistance to high salt stress and drought (9, 10). Spm, through its oxidation, generates β-alanine and pantothenate (vitamin B5) in the budding yeast *Saccharomyces cerevisiae*, and pantothenate is a biosynthetic precursor of coenzyme A and acyl carrier protein (11).

**Figure 1:**
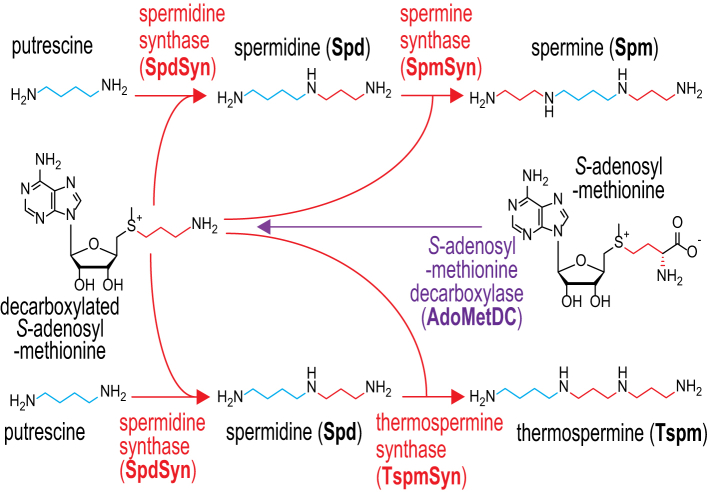
**Biosynthesis of spermidine, spermine and thermospermine in eukaryotes.** Aminobutyl groups are shown in *blue*, aminopropyl groups in *red*.

The Last Eukaryotic Common Ancestor is unlikely to have encoded SpmSyn or TspmSyn but very likely produced Spd from putrescine using AdoMetDC and the aminopropyltransferase Spd synthase (SpdSyn). Although different models have been proposed for the evolution of the first eukaryotic cell (Vosseburg 23), there appears to be a general consensus that eukaryotes evolved from a heterotrophic archaeal host cell and a bacterial endosymbiont that became the mitochondrion, as proposed by Sagan/Margulis (12). It is now thought that the host cell was related to an Asgard archaeon that may have been derived from Heimdallarchaeota (13) or a sister clade (14) and the bacterial endosymbiont was an α-proteobacterium (15). Thousands of genes from the protomitochondrion were transferred into the host nucleus by the process of endosymbiotic gene transfer (16, 17). A second endosymbiosis event also contributed to the genetic repertoire of many eukaryotes: the acquisition of a photosynthetic plastid by endosymbiosis of a cyanobacterium, which introduced thousands of genes into the eukaryotic host nucleus (18). The primary endosymbiosis of a cyanobacterium, of which the closest extant relative is *Gleoemargarita lithophora* (19) gave rise to the Archaeplastida (20). This large group of eukaryotes includes the red algae (Rhodophyta), Glaucophyta, green algae (Chlorophyta and Prasinodermophyta), and lands plants (Streptophyta).

Some amoeba-like eukaryotic cells subsequently took up either red or green algal endosymbionts in a process of secondary or serial endosymbiosis, followed by transfer of some of the algal nuclear genes into the host nucleus (21). Eukaryotic groups that evolved from independent secondary endosymbioses with a green alga include members of the Euglenozoa and Chlorarachniophyta, such as the photosynthetic/mixotrophic *Euglena gracilis* and *Chlorarachnion reptans*, respectively (22). Red algal endosymbioses occurred within the groups Cryptophyta, Haptophyta, Alveolata and Stramenopiles. A major group that was not systemically affected by endosymbiosis is the Amorphea, which includes the Amoebozoa and Opisthokonta. The latter includes the fungal and animal lineages. Other groups that were not systemically affected by endosymbiosis include the Heterolobosea in the Discoba, the Cercozoa, Foraminifera, Radiolaria, Telonemidae, Centrohelida and Kathablepharidae (22).

Cell growth and proliferation are dependent upon Spd in all eukaryotes, which furthermore is found throughout bacteria and archaea. Spd-derived Spm and Tspm are also found widely in eukaryotes, bacteria and archaea. The biosynthesis of these primordial molecules presents a tractable model for systematically studying the evolutionary processes that result in biosynthetic and enzymatic diversification. Emergence of eukaryotes from the fusion of an Asgard archaeon host cell and a α-proteobacterium represented a bottleneck in polyamine biosynthetic diversity relative to the contemporaneous prokaryotes. It is likely that the Last Eukaryotic Common Ancestor encoded an ornithine decarboxylase to produce the diamine putrescine, and an AdoMetDC and APT to convert putrescine to spermidine (Fig. 1) (23). Evolution of Spm or Tspm biosynthesis would therefore be dependent upon either *de novo* gene formation by, *e.g.,* gene duplication and neofunctionalization of the SpdSyn, or by gene acquisition from bacteria, viruses or other eukaryotes *via* horizontal or endosymbiotic gene transfer. Our goal was to identify, biochemically validate and map routes by which Spm and Tspm biosynthesis emerged during eukaryote evolution.

## Results and discussion

### Evolution of spermine biosynthesis in fungi

As a distinct lineage, the fungi are reported to have emerged between 1759 to 1078 million years ago (24). Only two SpmSyns have been reported in fungi. A SpmSyn (length 300 a.a.) that is similar but distinct from the corresponding SpdSyn has been identified in the budding yeast species *Saccharomyces cerevisiae* (Ascomycota, Saccharomycotina) (25). It has also been reported that a membrane protein-(APT-like domain) fusion protein (594 a.a.) is responsible for Spm biosynthesis in the filamentous rice blast fungus *Magnaporthe oryzae* (Ascomycota, Pezizomycotina) (26). These two SpmSyn proteins are very different. The *S. cerevisiae* SpmSyn is 49% identical at the amino acid sequence level to the *S. cerevisiae* SpdSyn, whereas the *S. cerevisiae* SpmSyn is only 30% identical to the C-terminal APT-like domain of the *M. oryzae* SpmSyn fusion protein.

Using a TBLASTN search of fungal genomes, we identified pairs of APT homologs similar to the *S. cerevisiae* Spd and Spm synthases (all proteins used in this study are listed in Tables S1 and S2). Among fungal genomes, these pairs of APTs are found only in the Saccharomycotina subphylum, *i.e.*, budding yeasts. The earliest branching lineage within the Saccharomycotina, that diverged approximately 400 million years ago (mya), is the Lipomycetaceae, followed by the Trigonopsidaceae, which diverged approximately 380 mya. (27). Pairs of APTs were identified in all Saccharomycotina lineages except the early branching Lipomyceteae and the Trigonopsidaceae, that encode only single (singleton) APTs. We decided to analyze the Spd/Spm synthase activity of pairs of APTs from the Diplodascaeae/Trichomonascaea clade, which diverged approx., 330 mya.; from the species *Yarrowia lipolytica* and *Wickerhamiella sorbophila*. We also selected a pair of APTs from *Debaryomyces hanseii* from the CUG-SER2 clade that diverged 230 mya. Singleton APTs from *Lipomyces starkeyi* (Lipomycetaceae) and *Tortispora caseinolytica* (Trigonopsidaceae) were also selected for analysis.

**Figure 2:**
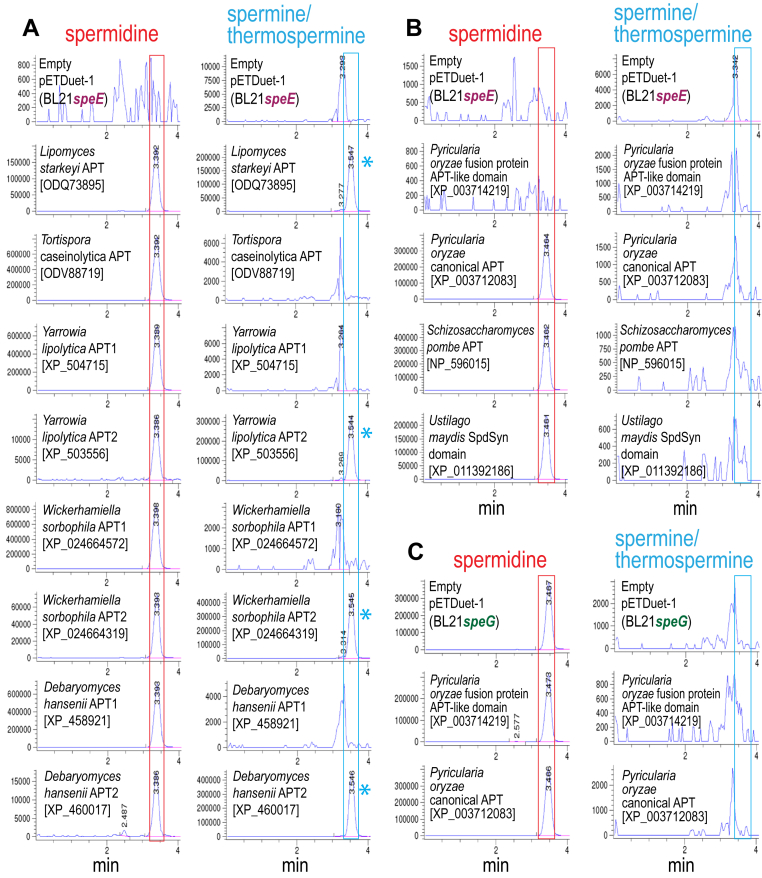
**LC-MS analysis of fungal aminopropytransferases expressed in *E. coli* BL21*speE* and BL21*speG*.** Polyamines from cell extracts were benzoylated and analyzed by LC-MS. The Extracted Ion Chromatograms (EICs) for tribenzoylated Spd (mass tolerance window 457.94:458.94), and tetrabenzoylated Spm/Tspm (619.02:620.02) are shown. Aminopropyltransferase homologs from the indicated species were expressed from pETDuet-1 in Spd devoid *E.coli* BL21*speE* (SpdSyn gene deletion) or in Spd replete BL21*speG* (Spd *N*-acetyltransferase gene deletion). Genbank protein accession numbers for each encoded protein are given in *brackets*. Spd peaks are highlighted by *red boxes*, and Spm/Tspm peaks by *blue boxes*, and the presence of a Spm/Tspm peak is indicated with a *blue asterisk*. APT, aminopropyltransferase. Cultures within each section (*A*–*C*) were grown and analyzed together, but *A*–*C* are independent experiments. The y-axis represents arbitrary units of ion intensity. EIC, extracted ion chromatogram.

The APT-encoding yeast genes were expressed from pETDuet-1 in the *E. coli* Spd devoid, SpdSyn gene deletion strain BL21*speE*, which contains putrescine. Cell extracts were benzoylated and analyzed by LC-MS, however, this system does not distinguish between Spm and same mass isomer Tspm. Figure 2*A* shows the Extracted Ion Chromatograms for tribenzoylated Spd and tetrabenzoylated Spm/Tspm. The singleton APT from *L. starkeyi* is clearly a bifunctional Spd/Spm synthase, producing approximately equal amounts of Spm and Spd when expressed in *E. coli*. This is the first identified case of a bifunctional Spd/Spm synthase in eukaryotes, *i.e.* in the absence of a dedicated SpdSyn encoded in the genome. In contrast, the singleton APT from *T. caseinolytica* produces only Spd, with no detectable Spm accumulation. Of the pairs of APTs from *Y. lipolytica*, *W. sorbophila* and *Debaryomyces hansenii*, one APT produces only spermidine, while the other produces primarily Spm with a much smaller amount of Spd remaining. This suggests that the SpmSyns can produce Spm from putrescine *via* Spd in the absence of a dedicated SpdSyn. To verify that the SpmSyns were not TspmSyns, we analyzed independently-grown BL21*speE* strains using LC-MS/MS, which unlike the LC-MS system, is able to discriminate chromatographically between the same mass isomers Spm and Tspm (Table 1). Although all the SpmSyns produced some detectable Tspm, the ratio of Spm to Tspm ranged from approximately 2.4 × 10^3^ to 1.0 for the *L. starkeyi* bifunctional APT to 1.2 × 10^4^ to 1.0 for the *W. sorbophila* SpmSyn. Using BLASTP, the closest eukaryotic homologs of the yeast SpmSyns are fungal SpdSyns, indicating that a SpdSyn gene duplication event gave rise to a neofunctionalized copy that exhibits a newly evolved SpmSyn activity but which retains SpdSyn activity. The gene duplication event probably occurred after 400 mya. but before 380 mya., *i.e.*, before the branching of the Diplodascaeae/Trichomonascaea lineage within the Saccharomycotina subphylum. Understanding fully the evolution of APT substrate specificity at the base of the Saccharomycotina evolutionary tree is complicated by the fact that the *L. starkeyi* APT is a bifunctional Spd/Spm synthase. For dedicated SpdSyn and SpmSyn gene pairs to have evolved from this enzyme would mean that a neofunctionalized copy of this gene would need to lose entirely SpmSyn activity, and the original APT would also need to greatly increase substrate preference for Spd over putrescine. A more parsimonious scenario for the evolution of specific SpmSyns is gene duplication of a dedicated SpdSyn such as the *T. caseinolytica* SpdSyn, followed by switch of substrate preference to include Spd as well as putrescine.

**Table 1:** LC-MS/MS analysis of spermine and thermospermine production by budding yeast SpmSyns in *E. coli* BL21*speE*

Aminopropyltransferase (species and [GenBank protein acc. no.] and size)	AUP 11.78 min (Spm)	AUP 11.05 min (Tspm)
Empty pETDuet-1	5.14 × 10^3^	N.D.
*Lipomyces starkeyi* [ODQ73895] 298 a.a.	2.71 × 10^8^	1.11 × 10^5^
*Yarrowia lipolytica* [XP_503556] 300 a.a.	6.51 × 10^8^	1.61 × 10^5^
*Wickerhamiella sorbophila* [XP_024664319] 283 a.a.	5.90 × 10^8^	4.92 × 10^4^
*Debaryomyces hansenii* [XP_460017] 314 a.a.	5.12 × 10^8^	6.18 × 10^4^

Cells extracts of *E. coli* BL21*speE* expressing aminopropyltransferase genes were benzoylated for analysis. The 497.200 daughter ion of the 619.228 parental ion for Spm and Tspm was used for quantification, after chromatographic separation, and peak validation was achieved using pure Spm and Tspm standards. AUP (area under the peak with elution time). All genes expressed from pETDuet-1. N.D., not detected. All strains were grown together in polyamine-free M9 medium and analyzed together.

The fungal phylum Ascomycota includes two major lineages, the subphyla Taphrinomycotina and Pezizomycotina (28). The model fission yeast *Schizosaccharomyces pombe* belongs to the Taphrinomycotina, and like all other Taphrinomycotina encodes only one APT. The *S. pombe* APT was expressed in *E. coli* BL21*speE* and produced only Spd (Fig. 2*B*). Previously, a gene disruption of the *S. pombe* APT was shown to deplete Spd (29), and the parental strain was found to contain a trace amount of Spm. When grown in polyamine-free medium, the relative amounts of Spm compared to Spd in *S. pombe* was less than 1%, whereas in *S. cerevisiae*, which has a dedicated SpmSyn, it was 15 to 20% (30). However, our heterologous expression of the *S. pombe* SpdSyn produced no detectable Spm. Phylogenetically diverse filamentous fungi were previously found to lack any detectable Spm (31). Recently, a gene deletion of an N-terminal membrane protein/C-terminal APT-like domain fusion protein was found to decrease but not deplete Spm in the filamentous Pezizomycotina species *Magnaporthe oryzae* (now known as *Pyricularia oryzae*) (26). We expressed the APT-like domain of the fusion protein (amino acids 254–594 of 594) and also the canonical APT-encoding gene from this species in BL21*speE* (Fig. 2*B*). The canonical APT homolog produced only Spd, and is thus a *bona fide* SpdSyn. However, the APT-like domain of the fusion protein did not produce Spd or Spm. We then expressed both *P. oryzae* genes in *E. coli* BL21*speG*, deleted for Spm/Spd *N*-acetyltransferase, which is spermidine replete, but no Spm was produced by the APT-like domain of the *P. oryzae* fusion protein (Fig. 2*C*). It is formally possible that the lack of detected SpmSyn activity for the *P. oryzae* fusion protein APT-like domain is due to expression problems. As a comparison, we also expressed the isolated APT domain (amino acids 1–288 of 769) of an N-terminal SpdSyn/C-terminal saccharopine reductase fusion protein from the filamentous Basidiomycota species *Ustilago maydis*, which produced Spd in BL21*speE* but no detectable Spm (Fig. 2*B*). Using BLASTP, we found that homologs of the *P. oryzae* membrane protein-(APT-like domain) fusion protein are found throughout the Fungi, including early diverging Chytridomycota, a pattern that is not consistent with the previously described distribution of spermine in fungi (31). When the *L. starkeyi* homolog of the *P. oryzae* membrane protein-(APT-like domain) fusion protein (GenBank protein acc. no. KAK9358249; 570 a.a.) is used to screen *L. starkeyi* proteins using PSIBLAST, the biochemically validated bifunctional Spd/Spm synthase of this yeast is not detected, suggesting that it is doubtful that the *P. oryzae* putative SpmSyn is related to any other polyamine biosynthetic APT. Outside of the Fungi, the only homologs of the *P. oryzae* membrane protein-(APT-like domain) fusion protein are bacterial proteins that have been experimentally shown to be uninvolved in Spd or Spm biosynthesis (32). We note that the original study identifying Spm in *P. oryzae* grew cells in rich medium that contains yeast extract and consequently, Spm (26).

### Evolution of spermine biosynthesis in the animal lineage

**Figure 3:**
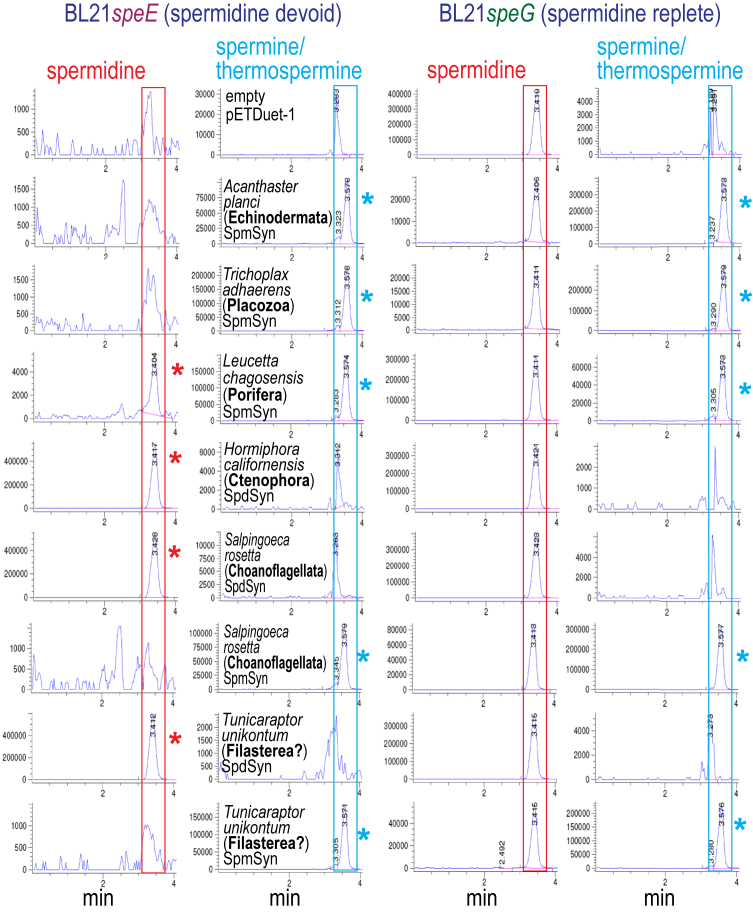
**LC-MS analysis of animal lineage SpdSyn and SpmSyn homologs expressed in *E. coli* BL21*speE* and BL21*speG*.** Polyamines from cell extracts were benzoylated and analyzed by LC-MS. The Extracted Ion Chromatograms (EICs) for tribenzoylated Spd (mass tolerance window 457.94:458.94), and tetrabenzoylated Spm/Tspm (619.02:620.02) are shown. Spd and Spm synthase homologs from the indicated species were expressed from pETDuet-1 in Spd devoid *E.coli* BL21*speE* (SpdSyn gene deletion) or in Spd replete BL21*speG* (Spd *N*-acetyltransferase gene deletion). Spd peaks are highlighted by *red boxes* and Spm/Tspm peaks by *blue boxes*, and the presence of a Spd peak in BL21*speE* is indicated with a *red asterisk* and a Spm/Tspm peak in BL21*speE* and BL21*speG* is indicated with a *blue asterisk*. Cultures within the BL21*speE* or BL21*speG* groups were grown and analyzed together but the BL21*speE* and BL21*speG* groups were independent experiments. The y-axis represents arbitrary units of ion intensity. EIC, extracted ion chromatogram.

Homologs of the human SpmSyn have been identified throughout the animal lineage (33), however, very few have been biochemically characterized. The X-ray crystal structure of the human SpmSyn (34) revealed that it contains an N-terminal domain that is very similar in structure but not amino acid sequence to the bacterial class 1b AdoMetDC (35) and a C-terminal bacterial-like APT domain, the whole fusion protein being reminiscent of bacterial AdoMetDC-SpdSyn fusion proteins (36). The animal SpmSyn can be easily distinguished from SpdSyns of the same species because of its extended class 1b AdoMetDC-derived N-terminal domain and the divergent, bacterial-like APT domain. It is an example of a gene that was likely originally acquired from bacteria *via* trans-kingdom horizontal gene transfer (33, 34). Although its APT domain functions as a SpmSyn in the human protein, it is not known if it was originally acquired from bacteria as an already functional SpmSyn or whether it was a SpdSyn domain that evolved within the animal lineage into a SpmSyn. We searched for homologs of the human SpmSyn in the basal animal (Metazoa) lineage phyla and sought to determine whether they exhibit Spm or Spd synthase activity. We also sought to identify the most basal pre-metazoan lineage that encodes a functional SpmSyn homolog. To identify SpmSyn activity, we expressed homologs of the human SpmSyn in Spd devoid *E. coli* BL21*speE*, and in a BL21*speG* strain, which is Spd replete (Fig. 3). We used LC-MS to detect Spd and Spm/Tspm (Fig. 3), and with independently grown cultures, we used LC-MS/MS to distinguish between Spm and Tspm (Table 2).

**Table 2:** LC-MS/MS analysis of spermine and thermospermine production by metazoan lineage aminopropyltransferases in *E. coli* BL21*speG*

Aminopropyltransferase (species, activity, size a.a.)	AUP 11.64 min (Spm)	AUP 10.96 min (Tspm)
Group 1		
Empty pETDuet-1	1.26 × 10^5^	N.D.
*Tunicaraptor unikontum* SpmSyn (380)	2.30 × 10^8^	N.D.
*Tunicaraptor unikontum* SpdSyn (296)	4.70 × 10^4^	N.D.
*Salpingoeca rosetta* SpmSyn (428)	4.86 × 10^8^	N.D.
*Salpingoeca rosetta* SpdSyn (290)	1.76 × 10^4^	N.D.
*Hormiphora californensis* SpdSyn (303)	1.38 × 10^5^	1.12 × 10^5^
*Leucetta chagosensis* SpmSyn (372)	1.07 × 10^8^	N.D.
*Trichoplax adhaerens* SpmSyn (359)	1.58 × 10^8^	N.D.
*Acantaster planci* SpmSyn (371)	9.98 × 10^8^	N.D.
Group 2		
Empty pETDuet-1	3.33 × 10^4^	N.D.
*Homo sapiens* SpmSyn (366)	1.22 × 10^8^	N.D.
*Chlamydomonas reinhardtii* TspmSyn (314)	N.D.	2.62 × 10^8^
*Orbicella faveolata* SpmSyn (371)	8.58 × 10^8^	4.89 × 10^4^
*Orbicella faveolata* TspmSyn (374)	4.11 × 10^6^	1.07 × 10^8^
*Pocillopora damicarnis* TspmSyn (364)	N.D.	7.66 × 10^6^
*Pocillopora damicarnis* SpmSyn (364)	8.00 × 10^8^	4.65 × 10^4^
*Pocillopora damicarnis* SpdSyn (288)	2.07 × 10^4^	N.D.

Cells extracts of *E. coli* BL21*speG* expressing aminopropyltransferase genes were benzoylated for analysis. The 497.200 daughter ion of the 619.228 parental ion for spermine (Spm) and thermospermine (Tspm) was used for quantification, after chromatographic separation and peak validation was achieved by using pure spermine and thermospermine. AUP (area under the peak with elution time). All genes expressed from pETDuet-1. SpmSyn, spermine synthase; TspmSyn, thermospermine synthase; SpdSyn, spermidine synthase. N.D., not detected. All strains from each group were grown in polyamine-free M9 medium and analyzed together. Groups 1 & 2 were grown and analyzed independently.

The Crown of Thorns starfish *Acanthaster planci* is a member of the Echinodermata, a basal phylum of the Bilateria. Its SpmSyn homolog produced Spm when expressed in *E. coli* BL21*speE*, with no detectable accumulation of Spd, and in BL21*speG*, although with approximately 4 times more Spm in BL21*speG* (Fig. 3). The placozoan *Trichoplax adhaerens*, which is a simple plate-like aggregation of cells, encodes a SpmSyn homolog that similarly produces Spm in BL21*speE* and BL21*speG*, with no accumulation of Spd in BL21*speE*. We found that species in the Porifera phylum (sponges) mostly do not encode homologs of the human SpmSyn, *e.g.*, *Amphimedon queenslandica*, *Oscarella lobularis* and *Oopsacas minuta*. However, some species do encode a SpmSyn homolog, such as *Sycon ciliatum* [XP_065184110; 384 aa] and *Dysidea avara* [XP_065882284; 358 aa]. The SpmSyn homolog from sponge *Leuchetta chagosensi*s, identified by TBLASTN from a GenBank transcriptome shotgun assembly, was expressed. It produces more Spm in BL21*speE* compared to BL21*speG*, with a trace of Spd accumulation in BL21*speE*. We noticed that although the sponge *Geodia barretti* does not encode a SpmSyn homolog, it does encode three homologs of the bacterial Chloroflexota TspmSyn [CAI8018185, 309 aa; CAI8036507, 304 aa; CAI8011339, 309 aa]. There is a bacterial class 1b AdoMetDC gene immediately upstream of the TspmSyn homolog in the *G. barretti* genome, and the encoded AdoMetDC is highly homologous to bacterial Chloroflexota proteins from the sponge microbiome. This may represent a case of horizontal transfer of a bacterial operon into a eukaryotic genome, as has been found in some fungi and amoebozoa (37).

The most basal phylum of the animal lineage is currently considered to be the Ctenophora phylum, or comb jellies (38). We were unable to identify SpmSyn homologs in any ctenophore genome or transcriptome. A typical SpdSyn homolog of the ctenophore *Hormiphora californensis* was analyzed in case it exhibited substrate flexibility but only Spd production was detected. LC-MS/MS analysis, which is more sensitive than the LC-MS analysis, revealed that the echinoderm, poriferan and placozoan SpmSyns are highly specific for Spm production, with no detectable Tspm production (Table 2). The ctenophore SpdSyn produced equally low trace levels of Spm and Tspm.

### Evolution of spermine biosynthesis in the premetazoan lineage

Single-celled Choanoflagellata are considered the sister group of animals, and phylogenetically, the closest relatives to animals (38). Homologs of the metazoan SpmSyn are found throughout the Choanoflagellata. A primitive form of clonal multicelled colonies is found in the choanoflagellate *Salpingoeca rosetta* (39), which is also being developed as a model choanoflagellate (40). We expressed a SpdSyn and a SpmSyn homolog from *S. rosetta*, in BL21*speE* and BL21*speG* (Fig. 3). The SpdSyn homolog produced only Spd in BL21*speE* with no accumulation of Spm, and no Spm was produced in BL21*speG*. In contrast, the SpmSyn homolog produced Spm in BL21*speE* without Spd accumulation, and produced more Spm in BL21*speG* than in BL21*speE*. LC-MS/MS analysis revealed that the *S. rosetta* SpdSyn produces a trace of Spm but no detectable Tspm, whereas the SpmSyn is highly specific with no detectable production of Tspm (Table 2).

It is thought that the Filasterea are immediately basal to the chaonoflagellates and animals (38), and we did not detect any SpmSyn homolog encoded by the filasterean *Capsaspora owcsarzaki* but it does encode a typical SpdSyn [XP_011270457, 353 aa]. However, using TBLASTN with transcriptome shotgun assemblies, we detected a SpmSyn and SpdSyn homolog encoded by *Tunicaraptor unikontum*, whose phylogenetic placement is not agreed but may be sister or basal to the Filasterea (41). Expression of the synthesized *T. unikontum* SpdSyn homolog produced Spd but no Spm in BL21*speE*, whereas expression of the SpmSyn homolog produced Spm with no detectable Spd (Fig. 3). Our LC-MS/MS analysis revealed that the *T. unikontum* SpmSyn is highly specific, with no detectable production of Tspm, and the SpdSyn produces a trace amount of Spm but not Tspm (Table 2). The single-celled Ichthyosporea are considered basal to the Filasterea, and we found that the genome of the ichthyosporean *Sphaeroforma arctica* does not encode a SpmSyn homolog but does encode a homolog (XP_014160118; 334 a.a.) of the Chloroflexota TspmSyn, which is analyzed in the TspmSyn section below. The general trend of the animal and pre-animal lineage SpmSyns was that expression of the homologs in Spd devoid BL21*speE* resulted in accumulation of Spm but not Spd, suggesting that the SpmSyn homolog synthesizes Spd from putrescine, and then efficiently synthesizes Spm from Spd.

### The animal SpmSyn evolved from a bacterial AdoMetDC-SpmSyn fusion protein

The progenitor of the metazoan SpmSyn was likely acquired from a bacterium by horizontal gene transfer in a single-celled organism before the emergence of the Choanoflagellata. The earliest branching example of a SpmSyn that we identified in the premetazoan lineage is the *T. unikontum* protein, and it is demonstrably a functional SpmSyn. We did not identify any homologous genes that exhibited only SpdSyn activity. This suggests that the bacterial fusion gene that was horizontally acquired was originally a SpmSyn, and since its acquisition predated the divergence of the Choanozoa (Choanoflagellatea + Metazoa), it was likely acquired more than 800 mya (38). At the amino acid sequence level, the human and *T.unikontum* SpmSyn proteins are 51% identical, primarily in the APT domain. Using these two sequences and other basal metazoan SpmSyns, we screened bacterial proteomes by BLASTP for the closest homologs of animal lineage SpmSyn. Closest matches were common to the different animal lineage SpmSyns, and exhibited between 30 to 32% identity. The bacterial sequences were mostly fusion proteins of a class 1b AdoMetDC and an aminopropyltransferase domain.

**Figure 4:**
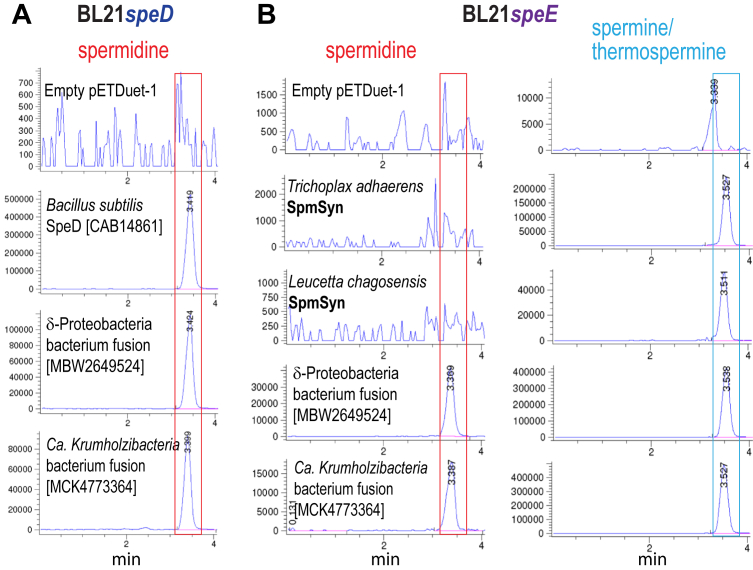
**LC-MS analysis of bacterial AdoMetDC-aminopropyltransferase fusion protein homologs expressed in *E. coli* BL21*speD* and BL21*speE*.** Polyamines from cell extracts were benzoylated and analyzed by LC-MS. The Extracted Ion Chromatograms (EICs) for tribenzoylated Spd (mass tolerance window 457.94:458.94), and tetrabenzoylated Spm/Tspm (619.02:620.02) are shown. Genbank protein accession numbers for each encoded protein are given in *brackets*. Spd peaks are highlighted by *red boxes*, and Spm/Tspm peaks by *blue boxes*. Cultures within the (*A*) BL21*speD* or (*B*) BL21*speE* groups were grown and analyzed together but the BL21*speD* and BL21*speE* groups were independent experiments. The y-axis represents arbitrary units of ion intensity. EIC, extracted ion chromatogram.

We selected a δ-proteobacterium and a *Ca. Krumolzibacteria* bacterium AdoMetDC-APT fusion protein for functional analysis. The AdoMetDC activity of the fusion proteins was assessed by expressing the fusion protein-encoding genes in *E. coli* BL21*speD*, an AdoMetDC (*speD*) gene deletion strain that is devoid of Spd. We used the *Bacillus subtilis* class 1b AdoMetDC-encoding gene as a positive comparison. The δ-proteobacterium [MBW2649524] and *Ca. Krumolzibacteria* bacterium [MCK4773364] fusion proteins each exhibited functional AdoMetDC activity, defined by the ability to produce Spd, detected by LC-MS (Fig. 4*A*). Potential SpmSyn activity was assessed by expression of each gene in Spd-devoid *E. coli* BL21*speE*. The placozoan *T. adhaerens* and sponge *L. chagosensis* SpmSyns were used as positive controls for SpmSyn activity. Unlike the placozoan and sponge SpmSyns, which produced Spm without accumulating Spd, the bacterial fusion proteins produced Spm and a detectable amount of Spd (Fig. 4*B*). This would explain why Spd is detected in the BL21*speD* strain expressing the bacterial fusion proteins, because not all Spd is converted to Spm. LC-MS/MS analysis revealed that the δ-proteobacterium fusion protein is a highly specific SpmSyn, with approx. 10,000-fold more Spm than Tspm produced, whereas the *Ca. Krumolzibacteria* bacterium fusion protein produces only about 60-fold more Spm than Tspm (Table 3). These data suggest that when the bacterial AdoMetDC-SpmSyn fusion protein was acquired early in the pre-animal lineage, it was likely already a SpmSyn, and did not evolve within the pre-animal lineage from a bacterial SpdSyn activity. However, the horizontally acquired bacterial fusion protein did lose the AdoMetDC function of the N-terminal domain, which nevertheless, because of its structural role in dimer formation, is essential for human SpmSyn activity (34).

**Table 3:** LC-MS/MS analysis of spermine and thermospermine production by bacterial and archaeplastida tetraamine synthases in *E. coli* BL21*speG*

Aminopropyltransferase species, [GenBank protein acc. no.] and size a.a.	AUP 11.63 min (Spm)	AUP 10.92 min (Tspm)
Empty pETDuet-1	9.22 × 10^3^	N.D.
Deltaproteobacterium [MBW2649524] 358	4.48 × 10^8^	5.51 × 10^4^
*Ca.* Krumholzibacterium [XP_ MCK4773364] 376	7.72 × 10^8^	1.14 × 10^7^
*Amborella trichopoda* [XP_020526675] 353	7.00 × 10^7^	N.D.
*Cryptomeria japonica* [GLJ38302] 395	3.64 × 10^7^	N.D.
*Selaginella moellendorffii* [XP_002993169] 322	4.66 × 10^8^	5.45 × 10^5^
*Cyanidiococcus yangmingshanensis* [KAF6001755] 332	2.42 × 10^8^	1.95 × 10^6^
*Porphyridium purpureum* [KAA8498204] 381	9.01 × 10^8^	1.84 × 10^5^
*Picozoa* sp. [S.R.A.] 374	3.67 × 10^4^	2.06 × 10^6^
*Dehalococcoidia* bacterium [HIM38018] 303	2.87 × 10^6^	1.92 × 10^8^
*Dehalococcoidia* bacterium [MSQ11180] 305	1.95 × 10^6^	2.92 × 10^8^
*Dehalococcoidia* bacterium [MSP79182] 305	1.37 × 10^6^	8.00 × 10^7^

Cells extracts of *E. coli* BL21*speG* expressing aminopropyltransferase genes were benzoylated for analysis. The 497.200 daughter ion of the 619.228 parental ion for Spm (spermine) and Tspm (thermospermine) was used for quantification, after chromatographic separation and peak validation was achieved by using pure spermine and thermospermine. AUP (area under the peak with elution time). All genes expressed from pETDuet-1. N.D., not detected. All strains were grown in polyamine-free M9 medium and analyzed together. S.R.A., Sequence Read Archive.

### Loss of spermine synthase in worms

We note that SpmSyn has been lost from almost all nematode species (round worms), although a homolog is present in *Soboliphyme baturini* [VDO95557; 377 a.a.]. In addition, SpdSyn and Spm synthase have been lost from all *Schistosoma* species (blood flukes). Within the Platyhelminthes phylum (flatworms), SpmSyn but not SpdSyn has been lost from all sequenced species, including all *Taenia* and *Echinococcu*s species (tapeworms), with the single exception of *Macrostomum lignano*. Loss of SpmSyn in parasitic worms appears to be an adaption to a parasitic lifestyle but it is also absent in free-living nematode worms such as *Caenorhabditis elegans*.

### TspmSyn evolved from SpmSyn in corals

**Figure 5:**
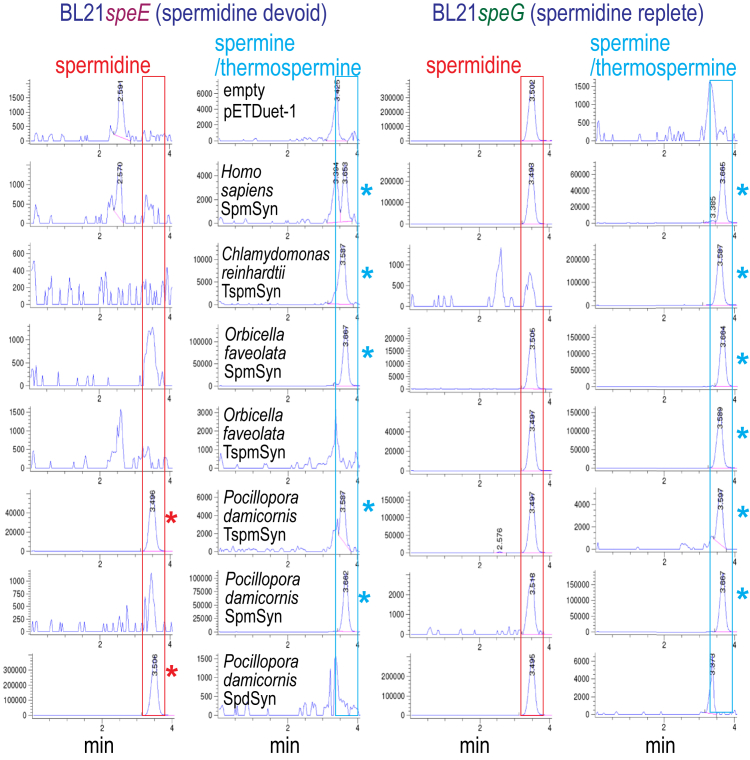
**LC-MS analysis of spermine/thermospermine production by coral aminopropyltransferase homologs expressed in *E. coli* BL21*speE* and BL21*speG*.** Polyamines from cell extracts were benzoylated and analyzed by LC-MS. The Extracted Ion Chromatograms (EICs) for tribenzoylated Spd (mass tolerance window 457.94:458.94), and tetrabenzoylated Spm/Tspm (619.02:620.02) are shown. Spd peaks are highlighted by *red boxes* and Spm/Tspm peaks by *blue boxes*, and the presence of a Spd peak in BL21*speE* is indicated with a *red asterisk* and a Spm/Tspm peak in BL21*speE* and BL21*speG* is indicated with a *blue asterisk*. Cultures within the BL21*speE* or BL21*speG* groups were grown and analyzed together but the BL21*speD* and BL21*speE* groups were independent experiments. The y-axis represents arbitrary units of ion intensity. EIC, extracted ion chromatogram;

The coral-containing Cnidaria phylum is unusual amongst the basal animal lineage because it contains species that encode two paralogs of SpmSyn that are only 45% identical (Fig. S1). We analyzed the two SpmSyn paralogs from the stony coral *Orbicella faveolata* and two SpmSyn paralogs and a SpdSyn homolog from another stony coral, *Pocillopora damicornis*. The human SpmSyn and *Chlamydomonas reinhartii* TspmSyn were used as positive controls. LC-MS analysis confirmed that the coral SpmSyn homologs produced Spm/Tspm (Fig. 5), in BL21*speE*, *i.e.*, the SpmSyn homologs can produce Spm/Tspm from putrescine. The SpdSyn homolog of *P. damicornis* produced only Spd and no Spm/Tspm. We then analyzed the coral SpmSyn homologs by LC-MS/MS, and found that one homolog from each species is specifically a SpmSyn, whereas the other homolog is a TspmSyn (Table 2). These are the first examples of homologs of the human SpmSyn exhibiting predominantly TspmSyn activity. The *P. damicornis* and *O. faveolata* SpmSyn and TspmSyn proteins are most similar to other cnidarian proteins. Coral SpmSyn-derived TspmSyns represent an independent invention of TspmSyn activity in eukaryotes.

### Evolution of spermine biosynthesis in land plants

**Figure 6:**
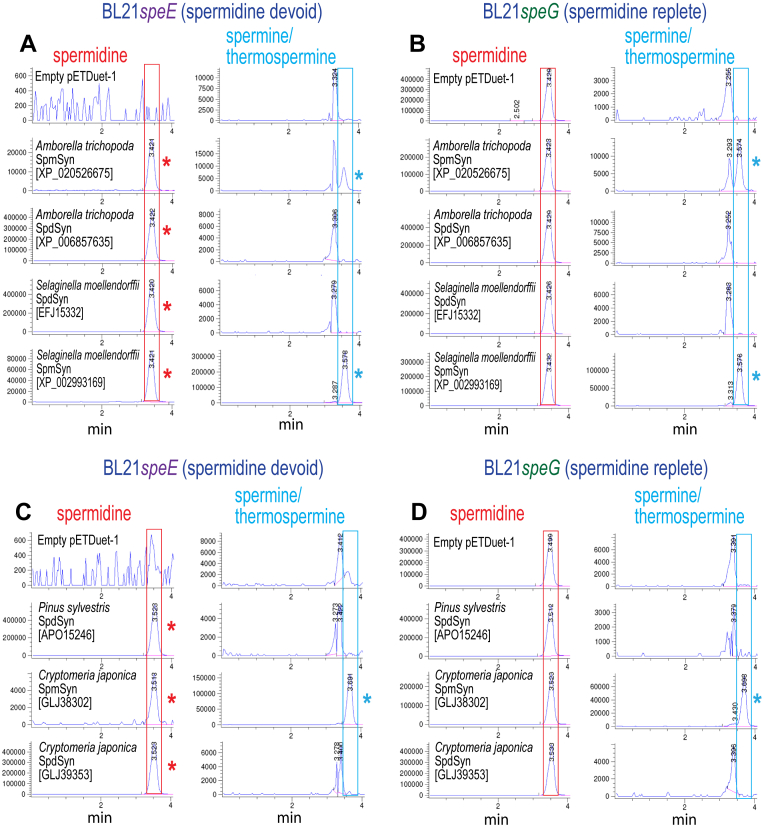
**LC-MS analysis of spermine/thermospermine production by plant aminopropyltransferase homologs expressed in *E. coli* BL21*speE* and BL21*speG*.** Polyamines from cell extracts were benzoylated and analyzed by LC-MS. The Extracted Ion Chromatograms (EICs) for tribenzoylated Spd (mass tolerance window 457.94:458.94), and tetrabenzoylated Spm/Tspm (619.02:620.02) are shown. Spd peaks are highlighted by *red boxes*, and Spm/Tspm peaks by *blue boxes*, and the presence of a Spd peak in BL21*speE* is indicated with a *red asterisk* and a Spm/Tspm peak in BL21*speE* and BL21*speG* is indicated with a *blue asterisk*. Genbank protein accession numbers for each encoded protein are given in *brackets*. Cultures within each section (*A*, *B*, *C* and *D*) were grown and analyzed together, but *A*, *B*, *C*, and *D* are independent experiments. The y-axis represents arbitrary units of ion intensity. EIC, extracted ion chromatogram; Spd, spermidine; Spm, spermine; Tspm, thermospermine.

The model flowering plant *A. thaliana* SpmSyn is homologous to SpdSyn, and likely emerged after gene duplication of SpdSyn and subsequent evolution of one copy to become a SpmSyn (42). There are two SpdSyn paralogs in *A. thaliana*, which exhibit 86% amino acid identity, whereas SpdSyn1 exhibits 66% identity with the single SpmSyn. Although potential SpmSyn homologs are found throughout flowering plants (angiosperms), few have been functionally characterized. It is thought that the plant *Amborella trichopoda* is sister to all other flowering plants (43, 44, 45), and may represent the earliest branching angiosperm. We identified a putative SpmSyn [XP_020526675; 353 aa] and SpdSyn [XP_006857635; 333 aa] from *A. trichopoda*. The genes encoding both proteins were expressed in *E. coli* BL21*speE* and BL21*speG* (Fig. 6). We showed previously that the *A. thaliana* SpmSyn produces about 15-fold more Spm in BL21*speE* than in BL21*speG* (46). The *A. trichopoda* SpdSyn produced a large amount of Spd in BL21*speE* but did not produce Spm/Tspm in either BL21*speE* or in BL21*speG* (Fig. 6, *A* and *B*). Spm was produced by the putative SpmSyn in both BL21*speE* and BL21*speG*, confirming protein XP_020526675 as a functional SpmSyn. Analysis of *A. trichopoda* SpmSyn expression in BL21*speG* by LC-MS/MS showed that the SpmSyn does not produce any detectable Tspm (Table 3).

The lineages giving rise to angiosperms and gymnosperms diverged approximately 360 mya (47). It was reported that the gymnosperm *Pinus sylvestris* has a dual function Spd/Spm synthase that supplies Spm for this species. The protein [APO15246; 345 aa] produced only a trace of Spm relative to Spd when the purified recombinant enzyme was analyzed (48). To determine whether this is a general feature of gymnosperm SpmSyns, we examined the function of the *P. sylvestris* Spd/Spm synthase, and compared this with a candidate SpdSyn [GLJ39353; 341 aa] and SpmSyn [GLJ38302; 395 aa] that we identified in the proteome of the gymnosperm *Cryptomeria japonica*. After expression of each gene in BL21*speE* and BL21*speG* and analysis by LC-MS, we found that the *P. silvestris* APT produced a large amount of Spd in BL21*speE*, but no detectable Spm/Tspm in either BL21*speE* or BL21*speG* (Fig. 6, *C* and *D*). In contrast, the *C. japonica* candidate SpmSyn produced considerably more Spm/Tspm than Spd in BL21*speE*, confirming that it is a *bona fide* SpmSyn, and the candidate SpdSyn produced only Spd. Analysis by LC-MS/MS confirmed that the *C. japonica* SpmSyn produces only Spm with no detectable Tspm (Table 3). It is likely that the *P. sylvestris* SpmSyn remains to be identified.

During the course of our study, Takahashi (49) discovered that the lycophyte *Selaginella moelendorffii* APT XP_002993169 [322 aa] exhibited SpmSyn activity. We identified a SpdSyn homolog [EFJ15332; 364 aa] from *S. moelendorffii* and expressed the candidate SpdSyn and *bona fide* SpmSyn in BL21*speE* and BL21*speG* (Fig. 6, *A* and *B*). From LC-MS analysis, we confirm that the SpmSyn is active in *E. coli*, producing approximately 5-fold more Spm than Spd in BL21*speE*, and also confirm that the candidate SpdSyn is a *bona fide* SpdSyn. Analysis by LC-MS/MS revealed that the *S. moelendorffii* SpmSyn produces approx. 1000-fold more Spm than Tspm when expressed in BL21*speG* (Table 3). The *S. moelendorffii* Spm and Spd synthases exhibit 63.5% amino acid identity, and the presence of a functional SpmSyn in this lycophyte species indicates that the SpdSyn-encoding gene duplication that generated SpmSyn occurred at least 440 mya in the plant lineage (47). We did not detect candidates for SpmSyn in the nonvascular bryophytes, suggesting that the emergence of SpmSyn in plants was coincident with the emergence of a vascular system.

### Evolution of thermospermine and spermine biosynthesis in basal archaeplastida

Archaeplastida are a eukaryotic monophyletic supergroup containing organisms with a photosynthetic plastid descended from a primary endosymbiosis of a eukaryotic cell with a cyanobacterium. The Archaeplastida consist of the Rhodophyta (red algae), Glaucophyta and green plant lineage (Chlorophyta and Prasinodermophyta green algae, and land plants) (20, 50). Functional homologs of the *A. thaliana* Acl5/TspmSyn are found throughout land plants and in chlorophyte algae (49). A notable exception is the important crop plant barley (*Hordeum vulgare*). A recent analysis of the barley genome found that Acl5/TspmSyn is absent (51), a notable discovery that we confirm. A previous polyamine analysis of barley grains found the presence of Spm but did not specifically assess for the presence of Tspm (52). Using the Genbank sequence read archive, we were able to identify blocks of homology to TspmSyn in other *Hordeum* species, including *H. secalinum*, *H. marinum* and *H. brevisubulatum* (results not shown). However, wild barley (*H. spontaneum*), the progenitor of domesticated barley (53), does not appear to encode Acl5/TspmSyn. This would suggest that barley TspmSyn was lost before domestication, and that Tspm is not essential for stem elongation in monocot barley, in contrast to dicot *A. thaliana* and cotton (8, 54). We found that domesticated barley encodes one SpdSyn and two SpmSyn homologs, whereas wild barley encodes one SpdSyn and one SpmSyn homolog (Fig. S2).

**Figure 7:**
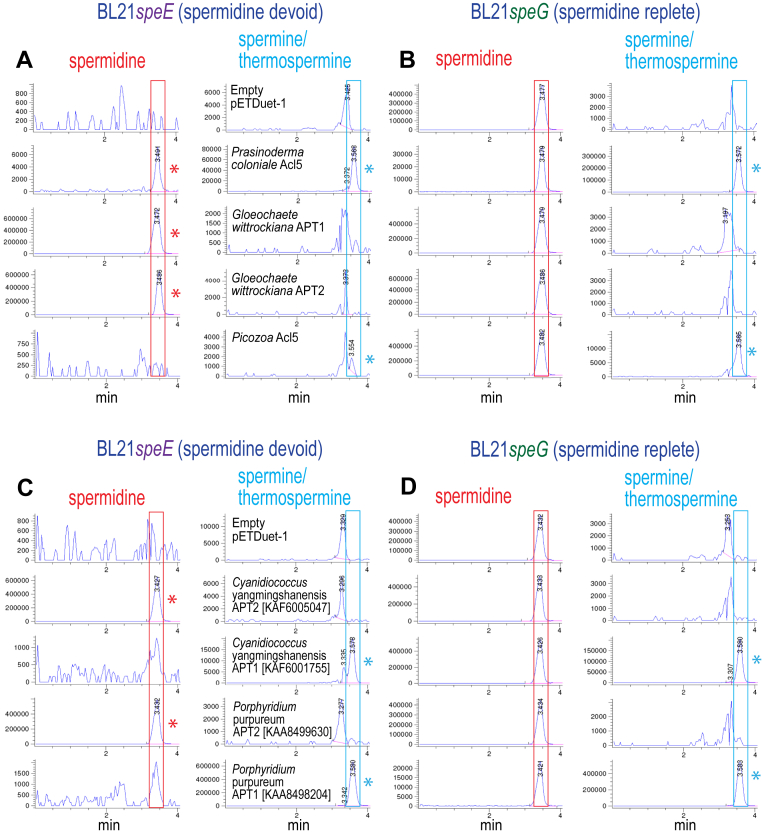
**LC-MS analysis of spermine/thermospermine production by aminopropyltransferase homologs from basal Archaeplastida expressed in *E. coli* BL21*speE* and BL21*speG*.** Polyamines from cell extracts were benzoylated and analyzed by LC-MS. The Extracted Ion Chromatograms (EICs) for tribenzoylated Spd (mass tolerance window 457.94:458.94), and tetrabenzoylated Spm/Tspm (619.02:620.02) are shown. Spd peaks are highlighted by *red boxes*, and Spm/Tspm peaks by *blue boxes*, and the presence of a Spd peak in BL21*speE* is indicated with a *red asterisk* and a Spm/Tspm peak in BL21*speE* and BL21*speG* is indicated with a *blue asterisk*. Genbank protein accession numbers for each encoded protein are given in *brackets*. APT, aminopropyltransferase. Cultures within sections *A* and *B*, or *C* and *D* were grown and analyzed together, but (*A* and *B*), and (*C* and *D*) are independent experiments. The y-axis represents arbitrary units of ion intensity. EIC, extracted ion chromatogram; Spd, spermidine; Spm, spermine; Tspm, thermospermine.

A putative Acl5/TspmSyn homolog from a Genbank transcript shotgun assembly derived from the prasinodermophyta alga *Prasinoderma coloniale* was identified, and we expressed the corresponding open reading frame (ORF) in *E. coli* BL21*speE* and BL21*speG*. LC-MS analysis showed that the *P. coloniale* APT was able to produce a large amount of Spm/Tspm in BL21*speE*, indicating that it produces Spm/Tspm from putrescine (Fig. 7, *A* and *B*). Analysis by LC-MS/MS of independently grown BL21*speG* cells expressing the *P. coloniale* APT confirmed that that the *P. coloniale* TspmSyn produces approx. 150-fold more Tspm than Spm (Table 4), and suggests that TspmSyn was present in the common ancestor of chlorophyte and prasinodermaphyte algae. The glaucophyte algae are thought to be basal to the green algae/plant lineage. We could identify only a SpdSyn homolog in the glaucophyte *Cyanophora paradoxa* using TBLASTN of transcripts at the Joint Genome Institute Genome Portal. However, we were able to identify a canonical SpdSyn (APT2) and typical Acl5/TspmSyn (APT1) homolog from the glaucophyte *Gloeochaete wittrockiana* using TBLASTN with the Genbank transcript shotgun assembly database. Both proteins, when expressed in BL21*speE* and BL21*speG* failed to produce Tspm/Spm but did produce Spd, and so both proteins, including the TspmSyn homolog, are SpdSyns (Fig. 7, *A* and *B*). The *G. wittrockiana* TspmSyn homolog exhibits 47% identity to the *A. thaliana* Acl5 Tspmsyn, and is the first example of a eukaryotic TspmSyn homolog to have evolved/devolved into a SpdSyn. It was noted previously that diverse glaucophyte algae contain only putrescine and Spd but not Spm/Tspm (55).

**Table 4:** LC-MS/MS analysis of spermine and thermospermine production by diverse eukaryotic thermospermine synthase homologs in *E. coli* BL21*speG*

Species [Genbank protein acc. no.] size a.a.	AUP 11.63 min (Spm)	AUP 10.92 min (Tspm)
Group 1		
Empty pETDuet-1	1.37 × 10^4^	N.D.
*Prasinoderma coloniale* [TSA] 323	6.08 × 10^5^	9.00 × 10^7^
*Sphaeroforma arctica* [XP_014160118] 334	4.26 × 10^6^	1.95 × 10^8^
*Aureococcus anophagefferens* [XP_009040305 ] 308	1.77 × 10^6^	1.03 × 10^8^
*Polarella glacialis* [CAE8606589 ] 322	1.23 × 10^6^	1.38 × 10^8^
*Cryptomonas paramecium* [TSA] 315	5.49 × 10^6^	2.10 × 10^8^
*Chlorarachnion reptans* [TSA] 329	4.33 × 10^5^	1.36 × 10^8^
*Planoprotostelium fungivorum* [PRP88509 ] 306	5.03 × 10^6^	5.73 × 10^7^
*Thecamonas trahens* [XP_013759236 ] 343	3.00 × 10^6^	1.07 × 10^7^
*Reticulomyxa filosa* [ETO33182] 376	1.82 × 10^5^	2.67 × 10^7^
Group 2		
Empty pETDuet-1	2.54 × 10^4^	N.D.
*Euglena gracilis* [TSA] 315	3.91 × 10^6^	6.57 × 10^7^
*Rhynchopus humris* [TSA] 344	4.05 × 10^5^	3.82 × 10^7^
*Percolomonas cosmopolitus* TsmSyn1 [TSA] 321	2.30 × 10^6^	3.80 × 10^7^
*Percolomonas cosmopolitus* TsmSyn2 [TSA] 322	5.79 × 10^6^	1.34 × 10^8^
*Balamuthia mandrillaris* [TSA] 358	9.08 × 10^5^	5.47 × 10^7^
*Vermistella antarctica* [TSA] 319	3.28 × 10^6^	1.20 × 10^8^
*Goniomonas avonlea* [TSA] 325	8.56 × 10^6^	1.29 × 10^8^
*Emiliania huxleyi* [TSA] 314	2.39 × 10^6^	2.51 × 10^8^

Cells extracts of *E. coli* BL21*speG* expressing thermospermine synthase genes were benzoylated for analysis. The 497.200 daughter ion of the 619.228 parental ion for spermine (Spm) and thermospermine (Tspm) was used for quantification, after chromatographic separation and peak validation was achieved by using pure spermine and thermospermine. All genes expressed from pETDuet-1. AUP, area under the peak with elution time; N.D., not detected; TSA, transcript shotgun assembly; a.a, amino acids. All strains from each group were grown in parallel in polyamine-free M9 medium and analyzed together. Groups 1 & 2 were grown and analyzed independently.

TspmSyn-like homologs are also encoded by red algae (Rhodophyta), and we expressed putative SpdSyn and TspmSyn homologs from the rhodophyte algae *Cyanidiococcus yangmingshanensis* (Cyanidiales) and *Porphyridium purpureum* (Porphyridiales), in BL21*speE* and BL21*speG*. LC-MS analysis indicated that Tspm/Spm was produced from putrescine in BL21*speE* by the rhodophyte TspmSyn homologs, and confirmed that the SpdSyn homologs are *bona fide* SpdSyn enzymes (Fig. 7, *C* and *D*). LC-MS/MS analysis revealed that the rhodophyte putative TspmSyns are in fact SpmSyns, with at least 100-fold more Spm than Tspm being accumulated (Table 3). Consistent with these results, members of the Cyanidiales and Porphyridiales were found to accumulate Spm but not Tspm (56). The rhodophyte SpmSyn appears to have evolved from a TspmSyn, rather than from a SpdSyn, and therefore the rhodophyte SpmSyn has evolved independently of the land plant SpmSyn. A close relative of the red algae is thought to be the plastid-lacking Picozoa (57). We searched the Genbank sequence read archive of Picozoa DNA sequences by TBLASTN to detect a potential TpsmSyn homolog. By tiling overlapping translated 151 bp DNA sequences, we were able to reconstruct a TspmSyn homolog, and expressed the full length ORF in *E. coli* BL21*speE* and BL21*speG* (Fig. 7, *A* and *B*). Tspm/Spm was accumulated in both BL21*speE* and BL21*speG*. LC-MS/MS analysis confirmed that the Picozoa sp. APT is a TspmSyn (Table 3).

When bacterial proteins are screened by BLASTP using diverse Archaeplastida TspmSyn amino acid sequences, the closest homologs are not cyanobacterial but Chloroflexota proteins. We synthesized three metagenome-derived Dehalococcoidia proteins that exhibit highest similarity to Archaeplastida TspmSyns, and expressed the relevant ORFs in *E. coli* BL21*speG*. LC-MS analysis confirmed robust Tspm/Spm production (Fig. 7), and LC-MS/MS analysis revealed that the proteins produced between approx. 60 to 150 times more Tspm than Spm (Table 3).

### Evolution of thermospermine biosynthesis in protist phyla

**Figure 8:**
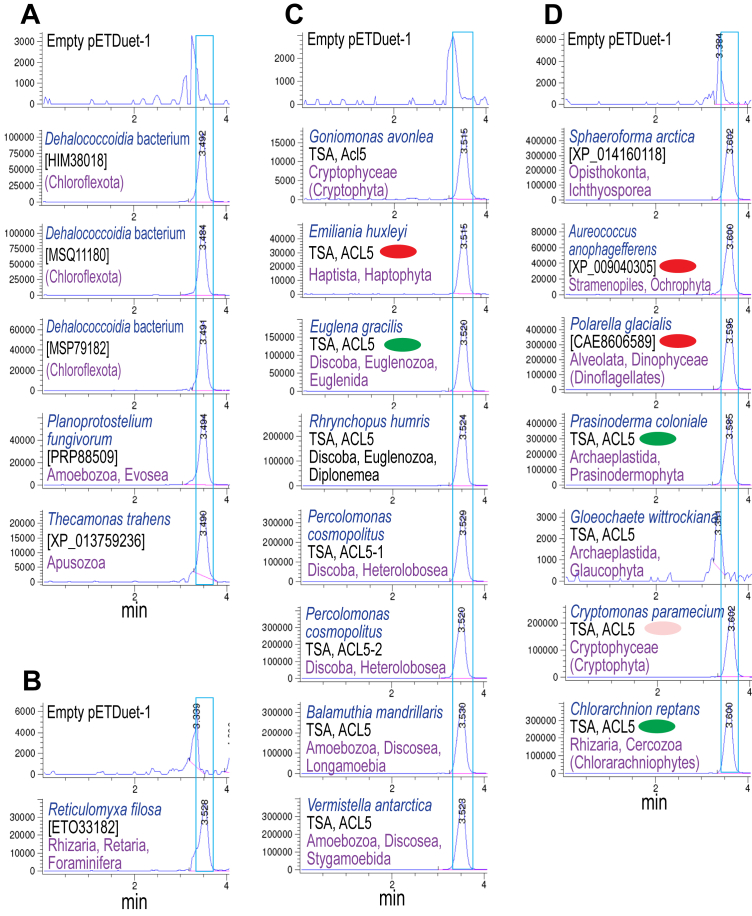
**LC-MS analysis of spermine/thermospermine production by protist aminopropyltransferase homologs expressed in *E. coli* BL21*speG*.** Polyamines from cell extracts were benzoylated and analyzed by LC-MS. The Extracted Ion Chromatograms (EICs) for tribenzoylated Spd (mass tolerance window 457.94:458.94), and tetrabenzoylated Spm/Tspm (619.02:620.02) are shown. Spm/Tspm peaks are highlighted by *blue boxes*. Genbank protein accession numbers for specific protein are given in *brackets*, where available. Acl5, TspmSyn homolog, TSA, transcriptome shotgun assembly. Cultures within each section (*A*–*D*) were grown and analyzed together, but *A*–*D* are independent experiments. *Green* and *red* ovoids denote presence of green or *red* algae-derived plastids, respectively. *Pink ovoid* denotes loss of previously present *red* alga-derived plastid. The y-axis represents arbitrary units of ion intensity. EIC, extracted ion chromatogram; Spm, spermine; Tspm, thermospermine; TspmSyn, thermospermine synthase.

A prominent feature of many protist lineages is that they contain a plastid that is derived from either a red or green alga (secondary endosymbiosis), and some have experienced multiple sequential endosymbioses (22, 24). In principle, some genes found in protists may therefore have originated with the endosymbiotic organism by endosymbiotic gene transfer to the host nucleus. We searched for TspmSyn homologs in all available protist lineages using BLASTP of the GenBank non-redundant protein sequence database, and TBLASTN of the transcriptome shotgun assembly database. Candidate genes were expressed in *E. coli* BL21*speG* from pETDuet-1, and we found accumulation of Tspm/Spm by LC-MS (Fig. 8) for all expressed TspmSyn homologs, and all were found by LC-MS/MS analysis to produce predominantly Tspm rather than Spm (Table 4). For lineages containing red algal-derived plastids, functional TspmSyns were confirmed for the photosynthetic haptophyte coccolithophore *Emiliania huxleyi* (58), the psychrophilic photosynthetic dinoflagellate *Polarella glacialis* (59), and the Stramenopile ochrophyte photosynthetic alga *Aureococcus anophagefferens* (60). We also demonstrated TspmSyn activity for genes from the secondarily plastid lacking cryptophyte *Cryptomonas paramecium*, that contains a red algal nucleomorph, a remnant nucleus of a red algal endosymbiont (61). We also demonstrated TspmSyn activity for a gene from the cryptophyte *Goniomonas avonlea*, which is thought to be ancestrally non-photosynthetic, and therefore unlikely to have acquired a TspmSyn by endosymbiotic gene transfer (62). Two photosynthetic lineages containing plastids derived from green algal endosymbionts were demonstrated to encode functional TpsmSyns (Fig. 8 and Table 4): the excavate flagellate *E. gracilis* (Discoba, Euglenozoa) (63), and the mixotrophic marine chlorarchniophyte *C. reptans* (Rhizaria, Cercozoa) (64).

A functional TspmSyn was confirmed (Fig. 8 and Table 4) from the ancestrally plastid lacking diplonemid excavate *Rhynchopus humris* (Discoba, Euglenozoa), and two functional paralogous TspmSyns, which exhibit only 53% amino acid identity (Fig. S3), were confirmed from the excavate free-living flagellate *Percolomonas cosmopolites* (Discoba, Heterolobosea). A functional TspmSyn was also confirmed for the foraminiferan *Reticulomyxa filosa* (Rhizaria, Retaria). The supergroup Amorphea contains the Amoebozoa lineage and the Opisthokonta (fungal and animal lineages) (65). There is no evidence of any endosymbiosis-derived plastid acquisition by this supergroup. We functionally confirmed TspmSyn activity for ORFs encoded by the Amoebozoa species *Vermistella antarctica* and *Balamuthia mandrillaris* (Amoebozoa, Discosea) and *Planoprotostellium fungivorum* (Amoebozoa, Evosea). The ichthyosporean *Sphaeoforma arctica* is one of the most basal single-celled groups in the pre-animal lineage (38) and we show that it encodes a functional TspmSyn. In addition, the nanoflagellates Apusomonodida are a sister group to the Opisthokonta (66), and we functionally confirmed that a TpsmSyn is encoded by the apusomonad *Thecamonas trahens*.

The presence or absence of TspmSyn homologs in protists is strongly correlated with the presence of algal-derived plastids, or an assumed loss of previously present plastids. In a large group such as the Amorphea, which has never had plastids, only relatively few examples of TspmSyn homologs could be identified amongst a very large number of sequenced genomes. TspmSyn homologs found in the Amorphea are likely to have been acquired from bacteria, viruses or endosymbiotic eukaryotes by horizontal gene transfer. In contrast, almost all sequenced genomes of the photosynthetic Haptista clade (haptophytes) in GenBank encode TspmSyn homologs: *Chrysochromulina tobini* [KOO53185; 319 a.a.], *Diacronema lutheri* [KAG8458031; 333 a.a.], and *Prymnesium parvum* [KAL151810; 311 a.a.], all of which exhibit 64 to 68% amino acid identity with the *E. huxleyi* TspmSyn. TspmSyns are found in groups that have never acquired algal endosymbionts, and so were never subjected to endosymbiotic gene transfer, such as the Amorphea. However, all of the TpsmSyns that we functionally identified, when used to screen bacterial proteins, are most similar to homologs from the Chloroflexota and not Cyanobacteriota, with the exception of the *T. trahens* TpsmSyn, which is most similar to cyanobacterial homologs.

### Conclusions and future directions

**Figure 9:**
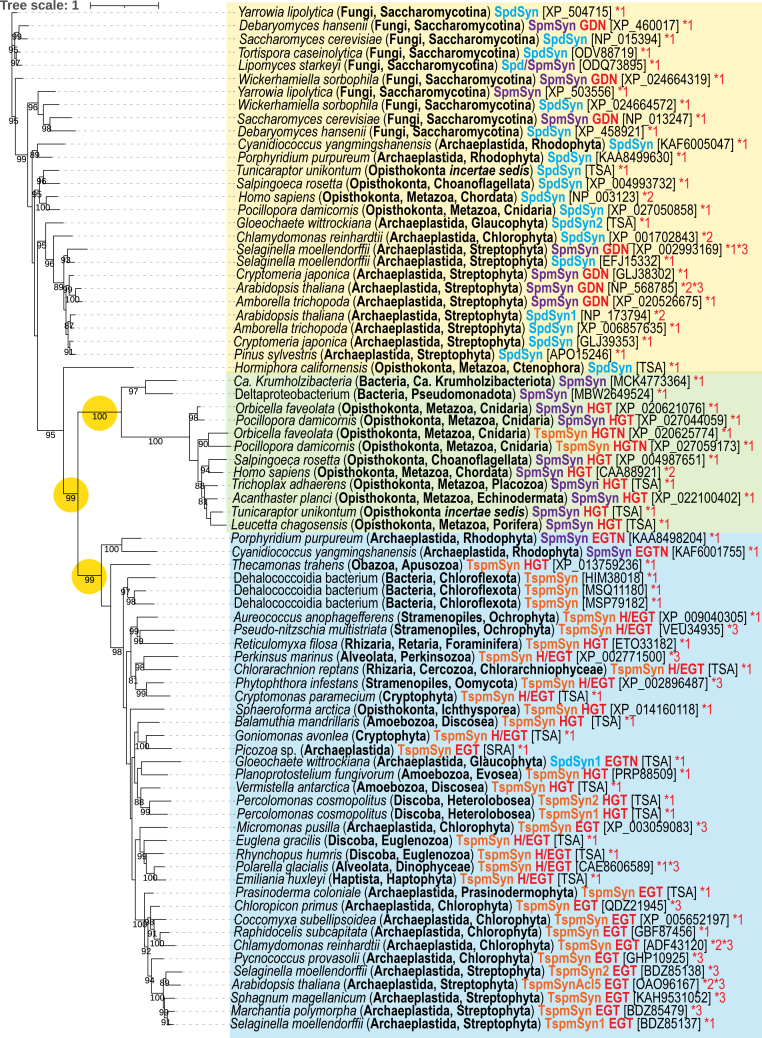
**Maximum Likelihood unrooted phylogenetic tree of functionally-characterized eukaryotic aminopropyltransferases.** SpdSyn, spermidine synthase; SpmSyn, spermine synthase; TspmSyn, thermospermine synthase; TSA, transcriptome shotgun assembly; SRA, sequence read archive. Likely origin of enzymes: GDN, gene duplication and neofunctionalization; HGT, horizontal gene transfer; HGTN, horizontal gene transfer and neofunctionalization; EGT, endosymbiotic gene transfer; EGTN, endosymbiotic gene transfer and neofunctionalization; H/EGT, probably endosymbiotic gene transfer but possibly horizontal gene transfer *via* viral transduction. Bootstrap supports of over 80% are shown on tree. Genbank protein accession numbers for specific protein are given in *brackets*, where available. ∗1, characterized in the current study; ∗2, (46), ∗3 (49). Numerical values represent percentage bootstrap support above 80% from 1000 ultrafast bootstrap analyses, and *yellow circles* denote major robustly supported clades. The scale bar represents the average number of amino acid substitutions per site. TspmSyn, thermospermine synthase; SpmSyn, spermine synthase.

We constructed a phylogenetic tree of functionally characterized eukaryotic aminopropyltransferases (Fig. 9). There are three strongly supported, and well distinguished clades: A, SpdSyn, and SpmSyn proteins that have evolved by gene duplication of SpdSyns; B, human-like SpmSyns that have evolved from a bacterial AdoMetDC-SpmSyn fusion protein; C, TspmSyns derived from a Chloroflexota TspmSyn. Sister to the TspmSyn domain are the Rhodophyta SpmSyns, and a TspmSyn homolog from the glaucophyte *G. wittrockiana* exhibits only SpdSyn activity. Human-like SpmSyns from corals exhibit TspmSyn activity.

Many algae and protists accumulate the symmetrical tetraamine norspermine (67), which is one methylene group shorter than Spm. In eukaryotes, it is thought that norspermine is made by the aminopropylation of norspermidine, that is produced from catabolism of Tspm by a Spm/Tspm oxidase (68). Biosynthesis of norspermine therefore requires aminopropylation of Spd by TspmSyn to form Tspm, the oxidation of Tspm to form norspermidine by a Spm/Tspm oxidase, and the aminopropylation of norspermidine by TspmSyn to form norspermine (69). The ability of TspmSyn to aminopropylate norspermidine to form norspermine appears to be an innate property of many bacterial TspmSyns (46). The seminal paper by Takahashi (49) revealed that different plant and algae TspmSyns exhibit differential preference for synthesizing norspermine *versus* Tspm. This property undoubtedly contributes to how much norspermine will be accumulated relative to Tspm. Nevertheless, even the TspmSyn homologs that prefer to aminopropylate norspermidine *versus* Spd must still produce Tspm in order to produce norspermine from the Tspm oxidation product norspermidine. The *A. thaliana* Acl5/TspmSyn is able to aminopropylate norspermidine to form norspermine (46, 49), however, norspermine is not found in *A. thaliana* because it does not encode a Tspm oxidase that produces norspermidine.

Further study is required to understand the dynamics of Tspm biosynthesis by TspmSyn, its catabolism to norspermidine, the aminopropylation of Nspd by TspmSyn to form norspermine, and the catabolism of norspermine to norspermidine by the Tspm oxidase (69). Another area of eukaryotic APT activity that requires further study is the APT domain-containing fusion proteins in diatoms, that are likely to be involved in long chain polyamine biosynthesis required for silica formation (70). For example, the diatom *Nitzschia inconspicua* encodes eight standalone APT homologs, and 32 fusion proteins containing an APT domain. One of the limitations of our current study is the identification of SpmSyns encoded by protist groups due to technical challenges. As another example, the pelagophyte *A. anophagefferens* encodes 12 APTs. We have not been able to address this issue here but it is a focus of our ongoing research. Finally, the physiological roles of Spm and Tspm in most organisms are unknown, and we hope that our current study will facilitate a better understanding of the biosynthesis of these evolutionarily ancient molecules.

## Experimental procedures

### Chemicals and reagents

Tspm (cat. no. Sc-472594B) was obtained from Santa Cruz Biotechnology. Spd (cat. no 85580), and Spm (85,605-1G) were obtained from Sigma Aldrich. The pETDuet-1 plasmid for expression in *E. coli* was obtained from Novagen. Synthetic genes with *E. coli*-optimized codons were purchased from GenScript. All proteins analyzed in this study are described in Tables S1 and S2.

### Bacterial strains, growth and gene expression

Generation of *E. coli* BL21 polyamine biosynthetic gene deletion strains was previously described: BL21*speD*, AdoMetDC-encoding gene deletion (32); BL21*speE*, SpdSyn-encoding gene deletion (71); and BL21*speG*, spermidine *N*-acetyltransferase-encoding gene deletion (72). Synthetic genes were ligated into pETDuet-1 with 5′-Nde1 and 3′-Xho1 sites, expressed from a phage T7 promoter, and selected with ampicillin. Cultures of *E. coli* were grown twice in 2 ml of liquid, polyamine-free M9 minimal medium, at 37 °C overnight. A 1.0 ml aliquot of culture was then centrifuged, the supernatant discarded, and cells resuspended in 10 ml M9 medium and grown at 37 °C to OD_600_ = 0.5. Gene expression from pETDuet-1 was induced by addition of 0.5 mM isopropyl-β-d-thiogalactopyranoside and cultures were maintained at 16 °C, overnight. Cells were then centrifuged, and polyamines extracted.

### Polyamine extraction and benzoylation reaction

Extraction of polyamines from *E. coli* cell extracts and benzoylation of polyamines was performed as described previously (73).

### LC-MS

Samples of benzoylated cell extract were run on an Agilent 1290 Infinity high performance liquid chromatography (HPLC) system fitted with an Eclipse XDB-C18 column (4.6 x 150 mm, 5 μm particle size), coupled to an Agilent 6130 quadrapole ESI mass spectrometer run in positive mode, employing a scan range of 100 to 1100 m/z. A flow rate of 0.5 ml/min at 20 °C was used for the liquid chromatography stage, with a 5 μl injection volume, employing a gradient elution with aqueous acetonitrile containing 0.1% formic acid.

### LC-MS/MS separation and quantification of tetrabenzoylated Spm and Tspm

HPLC conditions: reverse phase chromatography was performed using an ACE 3 C18-PFP 150 × 4.6 mm, 3 μm HPLC column (Mac-Mod). Column temperature, sample injection volume, and flow rate was set to 30 °C, 5 μl, and 0.8 ml/min respectively. HPLC conditions were: Solvent A: water with 0.1% formic acid (v/v), Optima LC/MS Grade. Solvent B: acetonitrile with 0.1% formic Acid (v/v), Optima LC/MS Grade: 40% B, 0 to 13 min; 5% B, 15 to 18 min; 95% B, 20 to 23 min; 40% B, 24 to 30 min. Total run time 30 min. Data was processed by SCIEX MultiQuant 3.0.3 software (AB Sciex) with relative quantification based on the peak area of each metabolite (https://sciex.com/products/software/multiquant-software). Targeted mass spectrometric analyses were performed on an AB Sciex QTRAP 6500+ mass spectrometer equipped with an ESI ion spray source. The ESI source was used in positive ion mode. Ion source conditions in the positive mode were: Ion Source Gas 1, 70 p.s.i.; Ion Source Gas 2, 65 p.s.i.; Curtain gas, 45 p.s.i.; ion spray voltage, 5500 V; and source temperature, 550 °C. Data acquisition was performed in multiple-reaction-monitoring mode. Three diagnostic multiple-reaction-monitoring transitions in the positive mode for tetrabenzoylated Spm (depending on individual runs, elution at 11.63–11.78 min) and Tspm (10.63–11.05 min) were obtained, and 3 MS ion transitions Q1/Q3, 619.228/497.2; 619.228/162 and 619.228/77 were monitored. The MS transition of Q1/Q3, 619.228/497.2, was used as the quantifier ion while 619.228/162 and 619.228/77 were used as the qualifier ions. The mass spectrometer was coupled to a Shimadzu HPLC (Nexera X2 LC-30AD), and was controlled by Analyst 1.7 software (https://sciex.com/products/software/analyst-software).

### Gene identification and phylogenetic analysis

Spd, Spm and Tpsm synthase homologs encoded by specific genomes were found by screening the NCBI non-redundant protein database with BLASTP or screening genomes, transcriptome shotgun assemblies or the sequence read archive by TBLASTN, using biochemically confirmed protein amino acid sequences. For construction of the Maximum Likelihood phylogenetic tree, the N- and C-terminal regions of Spd/Spm/Tspm synthases were trimmed to facilitate alignment. Amino acid sequence alignment was performed with MUSCLE (74), and the Maximum Likelihood phylogenetic tree construction was performed with IQ-TREE (75), using the “Auto” substitution model and 1000 ultrafast bootstraps analysis (76). The Maximum Likelihood phylogenetic tree was visualized with iTOL (77).

## Data availability

All data presented are contained within the article and Supporting Information.

## Supporting information

This article contains Supporting Information.

## Conflict of interest

The authors declare no conflicts of interest and no competing financial interests.
