# Supplementary material for: Spermine and thermospermine synthases emerged multiple times during eukaryote evolution
Source: J Biol Chem. 2025 Dec 9;302(1):111028. doi: 10.1016/j.jbc.2025.111028 (PMC12803844; doi:10.1016/j.jbc.2025.111028)
Supplement: Supporting information [file mmc1.pdf]

Supporting Information

Spermine and thermospermine synthases emerged multiple times during eukaryote evolution

Bin Li[1], Jue Liang[1], Hamid R. Baniasadi[1], Margaret A. Phillips[1] and Anthony J. Michael[1]*

[1]Department of Biochemistry, UT Southwestern Medical Center, Dallas, Texas, USA

*For correspondence: Anthony J. Michael, anthony.michael@utsouthwestern.edu

Table S1. Genbank protein accession no.

Species, APT activity, (size a.a.)	Protein acc. no.
**Metazoan lineage**	
Salpingoeca rosetta SpmSyn (428)	XP_004987651
Salpingoeca rosetta SpdSyn (290)	XP_004993732
Acantaster planci SpmSyn (371)	XP_022100402
Homo sapiens SpmSyn (368)	CAA88921
Orbicella faveolata SpmSyn (371)	XP_020621076
Orbicella faveolata TspmSyn (374)	XP_020625774
Pocillopora damicarnis TspmSyn (364)	XP_027059173
Pocillopora damicarnis SpmSyn (364)	XP_027044059
Pocillopora damicarnis SpdSyn (288)	XP_027050858
**Fungi**	
Lipomyces starkeyi Spd/SpmSyn (298)	ODQ73895
Tortispora caseinolytica SpdSyn (298)	ODV88719
Yarrowia lipolytica SpdSyn (292)	XP_504715
Yarrowia lipolytica SpmSyn (300)	XP_503556
Wickerhamiella sorbophila SpdSyn (293)	XP_024664572
Wickerhamiella sorbophila SpmSyn (283)	XP_024664319
Debaryomyces hansenii SpdSyn (297)	XP_458921
Debaryomyces hansenii SpmSyn (314)	XP_460017
Pyricularia oryzae APT-like fusion (254-594)	XP_003714219
Pyricularia oryzae SpdSyn (293)	XP_003712083
Schizosaccharomyces pombe SpdSyn (298)	NP_596015
Ustilago maydis SpdSyn fusion (1-288)	XP_011392186
**Plants**	
Amborella trichopoda SpdSyn (333)	XP_006857635
Amborella trichopoda SpmSyn (353)	XP_020526675
Pinus sylvestris SpdSyn (345)	APO15246
Cryptomeria japonica SpdSyn (341)	GLJ39353
Cryptomeria japonica SpmSyn (395)	GLJ38302
Selaginella moelendorffii SpdSyn (364)	EFJ15332
Selaginella moelendorffii SpmSyn (322)	XP_002993169
**Rhodophyte (red) and chlorophyte (green) algae**	
Cyanidiococcus yangmingshanensis SpdSyn (331)	KAF6005047
Cyanidiococcus yangmingshanensis SpmSyn (332)	KAF6001755
Porphyridium purpureum SpdSyn (295)	KAA8499630
Porphyridium purpureum SpmSyn (381)	KAA8498204
Chlamydomonas reinhardtii TspmSyn (314)	ADF43120
**Other phyla**	
Planostelium fungivorum TspSyn (306)	PRP88509
Thecamonas trahens TspmSyn (343)	XP_013759236
Reticulosa filosa TspmSyn (376)	ETO33182
Sphaeroforma arctica TspmSyn (334)	XP_014160118
Aureococcus anophagefferens TspmSyn (308)	XP_009040305
Polarella glacialis TspmSyn (322)	CAE8606589
**Bacteria**	
δ-Proteobacterium AdoMetDC-SpmSyn (358)	MBW2649524
Ca. Krumholzibacterium AdoMetDC-SpmSyn (376)	MCK4773364
Dehalococcoidia bacterium TspMsyn (303)	HIM38018
Dehalococcoidia bacterium TspmSyn (305)	MSQ11180
Dehalococcoidia bacterium TspmSyn (305)	MSP79182

SpmSyn, spermine synthase; TspmSyn, thermospermine synthase; AdoMetDC, *S*-adenosylmethionine decarboxylase

Table S2. Amino acid sequences of aminopropyltransferases generated from transcriptome shotgun assemblies and the sequence read archive.

>GHJI01006846.1 Genbank TSA: ***Trichoplax adhaerens* SpmSyn** evg1497205, translated from transcribed RNA sequence
MADDKVLHYMFDFRSIQTSLTPEAFIADASQFMKQKDFNLVCSSPLPHGQ
LLVWTGPDRSHAVIRLYQDINRITVDITKPLPDGKTITFSEDDEREIKSL
FQGNSKSYPAIQRCRDLDIYVPTCDNRLVEYDFDKVVVNVDTQFQNVKIC
HSPQYGNMLLLDDDPNLAESDIAYTKAITGNDTQDYTDKTVLILGGGDGG
ILNELLKQSPKYVTMVEIDQVVIDAAIKHLRGICENAMDSLEGPNYKVIV
EDCVPILKKYASEGKVFDYVINDLTAIPVSTTPQGSQWDFLRLILQLSMD
VLSETGRYFTQGNGANNVHALKLYEEQLQKLSRKVDFRKETVCVPSYLEF
WIFYEIWKV*

>GIYV01001903.1 Genbank TSA: ***Leucetta chagosensis* SpmSyn** breed sponge isolate calcarea TRINITY_DN8517_c0_g1_i2, translated from transcribed RNA sequence
MPLCQNILSFKLLPEHKDLLSNEETLSQLDQMLTSRGLGSRQTSVALPDG
RLVIYLDAERGRHATVRSQTTLPFPVLIIDVTQPVANETTAPAVSWESLN
ELYEQVKALFKCRKLSSLNFPPIHHGTEFERYFPSVDNRIAEYDFNRLVF
EADTPHQNVKILHSDQYGNTLVLDNDINLAESDIAYTKAITGSGRESYEG
KRVLILGGGDGGILNFLLPQSPKFVEMIEIDQVVIDQAVKHLRGICGSAM
DSLSGSNYKIRVEDCVPVLEQFAKDGVEFDYIINDLTAIPISTSPHGSFW
DFMRLILQLSFQVLAHNGKYFTQGNSVMAVNALAMYEGELAKLEPAVDFS
KEWVCVPSYQEMWVFYEMWRKS*

>GHXS01048829.1 Genbank TSA: ***Hormiphora californensis* SpdSyn** isolate 20161213-**T1** tx_DN15143_c5_g1_i3, translated from transcribed RNA sequence
MAKQWHTEENESEYPGQRFQLEEDNLIYETKSQYQTIRVFDHVKFGRVLS
LDGCVQGSTFDEFLYQEMLTYLPLCSTEDVSKVLVLGGGDGGVAREAAKD
PRVKSVIVVDIDEKVVETCRKFIPSMAASFDNPNVTLIIENAKTYLKNQL
DNSFNAVIIDLTDSPSSEDDSEPQFINSDLMRDIRRVLVDGGVVSQQDFA
PYSNNRLLSKSVDVFKATTTNICYGSVPVPCFPNGAIGICFGRVWKNGQD
HIDLSCPVWKISEECAVTMGLRCYYDRYHTASCTLPLDLKIWWREIVEKG
ENH*

>GIQG01041263.1 Genbank TSA: ***Tunicaraptor unikontum* SpmSyn** isolate Opistho-3 TRINITY_DN49007_c0_g1_i1, translated from transcribed RNA sequence
MIDDSTKSILGSGLRHMLMDFQIGEDVGTDAALDLALDALKSSCGLAETSRPAPSEDAAVVMVGDVKGAF
GTVTVVPSAALLTVDVTRTSGAALGDAALAQAGRDVEGALKEGTDGFRRSKLFPDIVRGGTLDPYVPTTT
NLLVQYDFDKTVVDEDSPYQNIKIMHSQQYGNVLLLDNDPNLAESDLSYTLAITGNGRENYEGKNVLILG
GGDGGILRVLREQKPKFVTMLEIDQLVIDYSVRHLRGICGDAMDELEGENYRVRVEDCIPALRDFAAQGV
EFDYIINDLTAIPISTSPQGEMWDFLRLILDLSFRVLSPTGKYFTQGNGANNTGALAMYEEQLRELCYAV
DFSKETVCVPSYLEMWVFYTVWKTAAEART*

>GIQG01038619.1 Genbank TSA: ***Tunicaraptor unikontum* SpdSyn** isolate Opistho-3 TRINITY_DN30173_c0_g1_i1, transcribed RNA sequence
MDALKSNSSKWFSEVQAEMWPGQCMSLEVEEVLYEGKSKFQDIILFKSKT
YGRVLVLDGVIQVTERDEFSYQEMLAHLPLFAHPNPEKVLVIGGGDGGIL
REVTKHECVKSVTLCDIDGDVIELSKKFLPFTACGYDDPRVTTHVGDGFK
FMAEHEGEFDVIITDSSDPVGPAQMLFEESYYQLMKKALRAGGVIASQGE
CAWLHKDLISGMLQFCGKNFGVTDYGYTCIPTYPSGQIGFMLCGLDAKTD
FRTPCRKPSEKMAESLRYYSTAMHRAAFVLPRQVEQQLYLE*

>HBDZ01012565.1 Genbank TSA: ***Prasinoderma coloniale* Acl5 (TspmSyn)**, MMETSP0806-doi:10.5281-zenodo.249982-Transcript-7283, translated from transcribed RNA sequence
MADAGGSKRLWLEEDIESDLRWSYTVKSILHTGKSEFQDVELVDTGPFGK
VLLLDGKLQSAERDEFVYHECLVHPALLAHPCPKTVFIAGGGEGSTAREV
LRHKTVEKCVMVDIDGVVVTFCKKHLDVNKEAFEDPRLELIIGDARSGLE
GYDGKFDVIVGDLADPVWGNPCYQLYTQAYYEMCKSKLNPGGVLVTQSGP
AGVLSCTEVYTVIHNTLKQVFPKVVPAAHHVPSFADTWGWQIAFCDEATG
AELGEAEIDARIAARVEGELAHYDGRCHAAITALSKSVRAALAKEERVYT
MESPVFIHGAGFNDNENVDRKGL*

>HBOJ01057359.1 Genbank TSA: ***Gloeochaete wittrockiana* SpdSyn**, TRINITY-DN6043-c0-g1-i1, transcribed RNA sequence (SpdSyn homolog)
MSIVDGWFREKGEDVCPGQGQSLQVEKVLFQGKSDFQDVLVFQSKTWGKV
LVLDGMIQVTERDEFSYQEMITHLPLCSHPNPEKVLVIGGGDGGVVRESI
KHPSVKEVTLCEIDGMVIDVAKQYFPALSCALTSGDPRVKVEVADGVAFL

DQKQNTYDVIIVDSSDPVGPAEGLFKLPFYEKLKKALRPGGIVCTQAECL
WLHLDLIKGLYKDIKEIFSSVEYAFVNTPTYPCGIIGFFICSNSGSCKSP
KRDPPLGLRYYSSAIHQASFVLPAFAQNLAQ*

>HBOJ01015314.1 Genbank TSA: ***Gloeochaete wittrockiana*** **TspmSyn-like SpdSyn**, TRINITY-DN12311-c3-g1-i1, transcribed RNA sequence
MARSYSNKIMQEEISEGLFGSYHLETIHFTGKSEIQEVEILTLTPYGLCL
VIDGKIQSTEKDEFIYHECMVHPALSIHPNPKSVFICGGGEGATAREALR
HNTVEKVHMVDIDTIVVDVSKKFLPKLHRGSFENPRFQLTISDARAHLEN
NDEKYDVIIIDLSDPLEDGPAYKLFSIEFYELCKRRLNENGLIVAQSGPA
GYTTSTTISMPVYASMKAVFKHVPTIRYHVPAFLDLYSFTIATDSDLVAH
NFYNPEGIDAVLEARIDARHETGATEEDVGAKVLKAYDSESHRELASLPK
WYRKFQSEANVTITDAAPRFYKAN*

Genbank Sequence Read Archive assembly and translation. **Picozoa sp. TpsmSyn.** Caution should be used with this exact amino acid sequence as it was manually assembled from translated short read DNA sequences, making assembly of a complete ORF challenging. The first 89 amino acids of the largest ORF (411 a.a.) were removed (red), and the expressed ORF initiated from an internal AUG.
MQEQQAHAGRPSAPGCHVLPAKLQTCALRCSRRHLAAMRSMHGACAVLRRGAK
PSHPLCTRVRTARTPSTKRGRAAPLLLAPRSTAAAKMSAGKWYHEDLGHGEMH
SMTFGAVLYSATTPFQKVEVLDTEPFGHVLMLDGVLNSAEKDEYIYHESLVQP
AMLAHPHPRTVFIGGGGEGATLREVLRHPSVEKAVMVDIDGDLVEVFRRDLPQ
YPQGAFDDPRTELIFDDAKKQLEEYDGLFDVIVLDLADPVEAGPAFPLWTREF
YEVLSPKMAPDGVLVTQAGPFSVNQVSEVCTPVAKTLGAVFPTVLTYGAHVPS
FGHTWAYNLAIKGGNHPASTYAERPAALVDRELAERLGAATAAALGWYDGLTH
AAMFSLPKPVRLALEAETRVVTLQTPLAFVAGAGLGQAKL*

>GGUN01041241.1 Genbank TSA: ***Goniomonas avonlea*** gonavon_41242 **TspmSyn** translated from transcribed RNA sequence
MCTEGKWILENLEEGLFHQFKFNKVLFTGESPYQTVEVLDTQTFGRCLML
DGLMQSAEIDEMIYHECLVHPAMVAHPNPKRVYIGGGGEGATLREVLKHK
SVEHVVMCDLDEKVVKLCQEHLRPHHKGALDPGCPRVELAFDDAKKYLED
HEGTWDVIILDLPDPVECGPAFTLYTQEFYAFCMSKLNPDGVLVTQSGPM
SVHSHNVVFTPINSTLRSVFPHVTAYGSYVPSFGHEWGYNIATKRPEEFA
SFSATPAEIDAKVEAKIDQTTQFGPLLFFDGISNQRIMSLSKITRKALAD
ETRVAKVDSPMTFNAGKGIAVTGAQ*

>HBTX01048407.1 Genbank TSA: ***Emiliania huxleyi*****,** **TspmSyn** TRINITY-DN3463-c0-g1-i1, transcribed RNA sequence
MSKWVEEEIETGLRVGYRLKKILDSSQSEFQTVDLVDLEPFGRTLMIDGL
IQSTQCDEFVYHEALVHPALLAHPNPRSVYIGGGGEGSTAREVLRHKSVE
RCVMVDIDADVVTFCKDHLPENAAAFADPRLELIIDDAKAVLEKEQNGFD
VIIMDLDDPLEGGPCYQLYTTEFYEMLRSKLNPGGIFVTQAGAAGIKQHR
LVWSPINSTLRAVFPAVTAYKHAVYSFLDEWGWHMASDSAELVAPLTPAE
VDARLKERLVGETTFLDGVSYPGLFSLSKTHRATLAAESLVMSREKGTFS
FMHNQGLCVAKAGK*

>GDJR01022779.1 Genbank TSA: ***Euglena gracilis*** **TspmSyn** comp22795_c0_seq2 transcribed RNA sequence
MLHIEETLEPGLRFLYEVEEVLHSTKTPFQTVAVVRTKAFGNALVI
DGLMQSADCDERIYHECLVHPAMLLHPHPRNVFIGGAGEGSTAREV
LKHNTVESCVMVDIDGDVVDFCKEVLPQNTQAFKDPRLTLIIDDAM
KQLEEATTLFDVIIMDLDDPLEGGPCYLLYTEEFYTMCRQKLAPNG
ILVTQCSSAGVKNYNLVFTPVNHTLKQVFPSVFGYSQHVYCFADAW
GFSLASNEPNATLPSAQEVDDRIAARGLGDLFFLDGISFGAIFALP
KYLRQALAKESRILTKAAKDYPKFMSCVQTIADKNEHKE*

>GJQB01007643.1 Genbank TSA: ***Rhynchopus humris*** **TspmSyn** TRINITY_DN22882_c0_g10128_i0, translated from transcribed RNA sequence.
MRSLTLSPPFPPQVYPRSHTPPTSTMSNIWVVEDLEQGLRFSYRIDEMLK
STQSKFQKVDVCDTTPFGKILIIDGLIQSSQLDEYIYHESLVHPALLAHP
NPKNVFIGGGGEGSTAREVLRHKTVEKCVMVDIDEKVVEFCREHLEENNA
AFKDPRLELIYDDCKIQLENSKIKYDVIIMDLDDPLEGGPCYWLYCQEFY
EMCKSKLNPGGILVTQASGAGVKQHTAVFSPVHNTISKVFPRTRGYCQAI
YSFADEWGYVIGYDDPETAKVTPEEIDARIAERVTGEMKFLDGDSYTGLF
CLNKTVKKNLAAETRVLSRDAPAVFLTHTTGVSSYTKDSTSTSN*

>HBGD01010243.1 Genbank TSA: **Percolomon*as cosmopolitus*****, Acl5-1 (TspmSyn1)** MMETSP0759-doi:10.5281-zenodo.249982-Transcript-8694, translated from transcribed RNA sequence

MNGTNKPTFLSMHNKWFIERCDENFFAVSSLTGLEYTGTTKFQKMDVVTL
GSFGRCLVLDDETQSSELDEMIYHESLVHPAMLMATNPERVLIAGGGEGA
TLREVLRHKSVKEAVMVDIDGECVEQCKKHLPQHHAGAFDDPRTTLIIDD
ANKIIQESDNESFDVIILDLADPVEGNPCFQLYAKSFYEMCKQKLRKGGV
LVTQSGPAGLVTFRNVFTPIHNTLKQVFKRVSPYSTYVPSFIDNYGFTVV
LKEDDSNLPDLTAVDPQWIDERINESISDPSILKHYDGISHRRMFNLPKQ
IRDGLSDEKRVISKDNFIFMH*

>HBLE01006561.1 Genbank TSA: ***Percolomonas cosmopolitus*, Acl5-2 (TspmSyn2)** MMETSP0758-doi:10.5281-zenodo.249982-Transcript-3001, translated from transcribed RNA sequence
MSEDTQAKDLSRHNFFYYEYVMEGFRGESKLLSIDFSGKTKFQNMEILEL
ESFGKCLVLDGEIQSAEKDEHIYHESLVQPAMLSHPNPKRVFIAGGGEGA
TAREVIKHPSVEECVMVDIDGDCVEYSKKLLKDHHKGALEHEKVKVIIDD
AKHYLEDIAEDNSFDVMILDLADPVKGGPCFQLYSDSWYELCKRKLTKNG
VLVTQSGCAGILTHDAILSPVHNTLKQVFSKVYPYSATIPSFCDDWGFNI
AFQSEEDAKALNDGQLDEKIEKRIEGGQTNMKFYDGISHTLLFALAKQIR
QTIAKEERVISKDNYVYVPCNQ*

>GISS01001711.1 Genbank TSA: ***Balamuthia mandrillaris* strain CDC-V039 TspmSyn** BamaA007183t1, translated from transcribed RNA sequence
MQNSNAKGMPPAAAAGKQPVRWYKEPVLPPDLYSLCVLKEVIHRQQSKYQ
TAEILNLSPWGVSLLLDGYMQSSTYDEYMYHESLVQPIMCSHPNPKTVFI
GGGGEGATAREVLRHKTVEKCVMVDIDQDVVEACKKHLPQHHKGAWNDKR
LELHYDDAKKILENYPEKFDVIILDLADPLEAGPCYLLYTVEFYEMCKTK
LNPGGLLVTQSGPGGALSMHQVFTPVAATLRKVFPHVVSYSTYIASFGDE
WGFNIASESEDVQNITAEEVDRRIAERIEGGAESLRYYDGTTHRHKVYPP
KHLSTAIAKETRTITADTPLFLQTVPTAEHMTAASAPTDVEEGAHSPPIS
ASPVAAAQ*

>GELU01003479.1 Genbank TSA: Vermistella Antarctica TspmSyn Contig_1994_1084_reads_1404_bases translated from transcribed RNA sequence
MSLVPGKNKWLVETISDDFVVINTMLTIYHTSQSKFQKVDVIETGAFGRC
LVLDDKMQSAEADERLYHESLVQPSMLLHPNPKTVFVGGGGEGATIREIL
RHKSVEKCVMADIDGETVQVCKEHLKQHHDGAFEDPRLELVIDCAKKTLI
ESPISFDVIILDLADPVQGGPCYLLYTKSFYELAKSKLNPGGVLVTQSGP
AGLTTHTEVLTPVNRTIREVFGEDNVHSYMTHIPSFTDLYAFTLARTEGL
ANPTSFEPEQVDKTMKERLPEWDSFHYDGLTHRNMFTLPKYARLSLAKEQ
HVITEDDPAFIYAGDRSDA*

>HBKS01038483.1 Genbank TSA: ***Cryptomonas paramecium*, TspmSyn** MMETSP0038-doi:10.5281-zenodo.249982-Transcript-44631, translated from transcribed RNA sequence
MSLQSEHRCNGFYYEEMVTTTLFLKSELKSIGFNSESKFQRLQIVETEPF
GKTLVIDDHTQSSALDEHVYHEALVHPVLLAHGNPKTVFIGGGGEGATAR
EILRWKSVEKLVMCDIDEIACKTCREQLPEWSEGVYEDPRFEIHYEDAFA
FLQNWKGPKFDVIIMDICDPIEAGPGVVLYFQEFYQSIARDKMTEGGVFV
TQSGACGFLNYHEVFTTIHSTCRSSFEHVHAYSVDVPSFGCPWGYNMCYN
SCAWPDAPSWDPAEVDRRLAAALGEEAAAALHHYDGVSHRALFHVPKMVR
KAMAKETRVMTKDTP*

>HBKK01026129.1 Genbank TSA: ***Chlorarachnion reptans*, TspmSyn** MMETSP0109-doi:10.5281-zenodo.249982-Transcript-33512, translated from transcribed RNA sequence
MAAAGDERKAAMSLGLTLTEAQAPGLKRQIMVEEVFFQAKSQFQENAVVR
TAAFGRALFMDKQIQSSEIDEFIYHESLVGPAMTIHPNPKRVFIGGGGEF
ATAREVLKHKGVEECVMVDIDEEACKICIEKLPTWHDGCVSDKRLEVVYG
DAMKYMFESKKKFDVVIMDIADPVEAGPGYKLYTKEFYEHVAKNKLNPGG
VIVTQSTAADIMQCRKSYATIHNTLKSAFDNVFGYSAEIISFGGAWGFNI
AWNKHGSLPDSPFGLSVGEVDKRIKEKYSADAKETKFYDGVTHQHLFHLP
KYLRKALADCTAVSTIENPCYMGYGALET*

```
                    *        20         *        40         *        60         *        80
HsSpmSyn   : MPG---AAARHSTLDFML--GAKADG---ETILKGLQSIFQ--------EQGMAESVHTWQ------------DHGYLAT :  52
SrSpmSyn   : MSEGSEVAVRGLHASPCL--AAHNGSDRVHHILIDLRASKQRMCDLSMTATRMREALQAVEGLALDHVRDCNSDNAAVAF :  78
PdTspmSyn  : -------MAKHSIFDFQS--PDADLKD--DVLRSRLFEILRKY------ASGPVISTELED------------GKSCLFI :  51
OfSpmSyn   : ------MVIYHSIFDFQA--PETDLRN--DDVRSQLFKILRKYH-----ATGPVVSTELED------------GKSFLFV :  53
PdSpmSyn   : ------MSVNHTLLDFRVTPDP--SGNWMVSVSEKMGRIIEESFK----IEKPEFSSP------------------TTML :  50
OfTspmSyn  : ------MSVKQTLLDFNVTLPAPLDDKMMTYLKRKVTEELQKTFG----TEDS-ISQPRPN----------------TMLL :  54
HsSpdSyn   : -------------------------------------------------------------------------------- :   -
PdSpdSyn   : -------------------------------------------------------------------------------- :   -
SrSpdSyn   : -------------------------------------------------------------------------------- :   -

                    *       100         *       120         *       140         *       160
HsSpmSyn   : YTNKNGSFANLRIYPHGLVLLDLQS--YDGDAQGKEE----IDSILNKVEER---------------------------- :  98
SrSpmSyn   : LCSNAGSFGTVRALATGLCTVDISLSLTAGEEPITEEQVAELKSTLQQVVDETCGGSEASAEASTATSTRSSSNTVPSSA : 158
PdTspmSyn  : FVGENGGHWILRSFSTGLITLDVMQ--YGKEE-------ILSKDTLKSIEKEI--------------------------- :  95
OfSpmSyn   : FIGKDGGHWTLRSFSSGLVTLDVMQ--YGKEESGEEL--AVSKDQLKNLAKEI--------------------------- : 102
PdSpmSyn   : FAGQKGIQCTVQLFPDGLVTLDVVQ--YINNISSE---QILKRSDYIDLRDKVD-------------------------- :  99
OfTspmSyn  : FFDKTETQCTVRIFPDGLVTLDIVQ--YVGDNKSDLY-RTWTKKDIVELRDRMN-------------------------- : 105
HsSpdSyn   : -------------------------------------------------------------------------------- :   -
PdSpdSyn   : -------------------------------------------------------------------------------- :   -
SrSpdSyn   : -------------------------------------------------------------------------------- :   -

                    *       180         *       200         *       220         *       240
HsSpmSyn   : -MKELSQD----STGRVKRLPPIVRGGAIDRYWPTADGRLVEYDIDEVVYDEDSPYQNIKILHSKQFGNILILSGDVNLA : 173
SrSpmSyn   : QHNEPSEDNETQTSVSAKVFPPIVRGGAFDPYVPSADGLLIQYDFDKTVFHEQSPYQDVKIMHSRQFGNILLLDDDVNLG : 238
PdTspmSyn  : -CKVLSAE-------KSLHLPPINRGAKVNTYYPTVDDLLIQYDFDRIEFETNSPYQNIKILHSQQFGNILILDDDINLA : 167
OfSpmSyn   : -FDVLSPL-------KSLLLPPISRGADMNTYYPTEDCLLIQYDFDRLIYETNSPHQNIKVLHSPQFGNMLILDGDINLA : 174
PdSpmSyn   : --EALSCS-------KAHFIPTIHRGREILRYHDTSDGRMKEYDFDKEVFKQKSDYQDVWIAHSSRYGNMLFLDLEEMLA : 170
OfTspmSyn  : --TSLSCS-------IARYILPITRGRAIRCYRETTDDRIIEYDFDRVVSSEHSPYQHIQVIHSPQFGNMLILDEIEMIA : 176
HsSpdSyn   : ----MEPG---PDGPAASGPAAIREGWFRETC-SLWPGQALSLQVEQLLHHRRSRYQDILVFRSKTYGNVLVLDGVIQCT :  72
PdSpdSyn   : ----MN---------------RIQNGWFSEIN-DQWPGQALSLEVEEIIFEGKSKYQDILVFKSKHHGKVLVLDGVIQCT :  60
SrSpdSyn   : ----MD---------------AMKKGWFSELSPEMWPGQCMSLEYEKVLEDIKSDYQHIVVFKSKTYGNVLVLDGVIQIT :  61

                    *       260         *       280         *       300         *       320
HsSpmSyn   : ESD-LAYTRAIMGSGKEDYTG-KDVLILGGGDGGILCEIVKL-KPKMVTMVEIDQMVIDGCKKYMR------------KT : 238
SrSpmSyn   : ESD-IAYTKAIMGSGSIDYTG-KDVLILGGGDGGILREIVKL-NPKMVTMVEIDQVVIDAAKEHLR------------GI : 303
PdTspmSyn  : ESD-FIYTKTITGSGREVFKD-KTILILGGGDGGILNELLQC-SPEFVTMVDIDEDVINCAIKYLR------------GI : 232
OfSpmSyn   : ESD-IMYTKTITGSGREDFKD-KTILILGGGDGGILNELLQY-SPKFVTMVDIDEEVVNCAIKYLR------------GI : 239
PdSpmSyn   : ESD-IDYTKALLGNGRESYKD-KDVLILGGGDGGVLNELLKE-SPKFVTMVEIDKVVVDASINHLR------------GI : 235
OfTspmSyn  : ESD-LVYTQALMGNGRADYKD-KSVLILGGGDGGVLHELLKE-NPRSVVMVEIDKGVIDASRKHLR------------AI : 241
HsSpdSyn   : ERDEFSYQEMIANLPLCSHPNPRKVLIIGGGDGGVLREVVKHPSVESVVQCEIDEDVIQVSKKFLPGMAIGYSSSKLTLH : 152
PdSpdSyn   : DRDEFSYQEMITFLPLNSHPCPRKVLVVGGGDGGVIREVSKHPAVESIVQCEIDEEVIEVCKKHLPNMAEGFSSPKLTQY : 140
SrSpdSyn   : ERDECGYQEMITHLPMFCHPNPERVLIIGGGDGGVLREVLRHPCVKEVTQCEIDGKVIELSKKHLPGIASCFDDPRANVI : 141

                    *       340         *       360         *       380         *       400
HsSpmSyn   : CGDVLDNLKGDC--YQVLIEDCIPVL--KRYAKEGREFDYVINDLT--AVPISTSPEEDSTWEFLRLILDLSMKVLKQDG : 312
SrSpmSyn   : CGDAMDSLTGDN--YEVLVKDCIPEL--ERWGKEGRAFDVIINDLT--AIPVTHSPK-GSDWDFLKLVLQLSMTVLRQGG : 376
PdTspmSyn  : CGNALDNLSGEH--HKVHIGDCVKYL--EQCIEEGRTFDYVINDLT--AIPVTKEPI-GSLWEFLRLILDMSMKVLSPSG : 305
OfSpmSyn   : CGDSLDNLSGEH--HKVHIDDCVKQL--EQYIKEGRTFDYVINDLT--AIPVTKEPR-GSLWDFLRLILDLSLKVLSPNG : 312
PdSpmSyn   : CGSSMDSYKGHN--FEIRIDDCVKAM--KEYVKSGKTFDYVINDLT--DIPV-SVSLHHSHWDFVREILNLTLQVLKPEG : 308
OfTspmSyn  : CGDSLDNLRGKN--YEIIIDDCAKVM--REFVREKKAFDFVINDLT--DIVIRSEEQDGSYLEFQREIQDLSIRLLSPRG : 315
HsSpdSyn   : VGDGFEFMKQNQDAFDVIITDSSDPMGPAESLFKESYYQLMKTALKEDGVLCCQGECQWLHLDLIKEMRQFCQSLFPVVA : 232
PdSpdSyn   : VGDGFDYMKKHENEFDVIITDSSDPVGPAESLFQESYYELMKKALKPDGLLCCQGECLWLHIPLIKSVLDFCRRLFPSVS : 220
SrSpdSyn   : VGDGFKFMKEHEGSFDVIITDSSDPIGPASSLFEKEYYHLMKTALRPGGIVCSQGECVWLHLDLIKSMQDFCKELYPTVG : 221

                    *       420         *       440         *       460         *
HsSpmSyn   : KYFTQG-NCVNLTEALSLYEEQLGRLYCP----------VEFSKEIVCVPSYLELWVFYTVWKKAKP--- : 368
SrSpmSyn   : RYLTQG-NSFNMPQALAMYEQQLTTLPCK----------LSFTKETVTVPSYHEQWVFYNVLE------- : 428
PdTspmSyn  : KYFTQG-NSYNKPDALLMYEQQLTKLSCP----------VQFRKEDVFVPSYHEKWVFYEIWKTNESTLS : 364
OfSpmSyn   : KYFTQG-NSFNKPEALLMYEQQLAKLSCP----------VEYRKEGAFVPSYHEKWVFYEIRKTDQSAKS : 371
PdSpmSyn   : KFFAQGTNGATCKKQIEQYEQQLMNLCYP----------VEFRREFAFVPSFEESWTFYEVWRKRT---- : 364
OfTspmSyn  : KYFVQGGNGPNMRESLSLYEEQLAKLYCP----------VEFRKDFAFVPSFLELWTFYEIWKKDTQDK- : 374
HsSpdSyn   : YAYCTIPTYPSGQIGFMLCSKNPSTNFQEPVQPLTQQQVAQMQLKYYNSDVHRAAFVLPEFARKALNDVS : 302
PdSpdSyn   : YGYTTIPTYPSGQIGFILCSKNKETKFDNPVTEFTEDQLEKEQLRYYNADVHRTAFVLPQFAKKALLI-- : 288
SrSpdSyn   : YAYATVPTYPSGQIGFTLCSLDDSADLAHPCRTLTQEQVQEMGLQYYNTDVHTAAFVLPQFARKKLASD- : 290
```

Figure S1. Amino acid sequence alignment of coral aminopropyltransferases. HsSpmSyn, *Homo sapiens* SpmSyn [CAA88921]; SrSpmSyn, *Salpingoeca rosetta* SpmSyn [XP_004987651]; PdTspmSyn, *Pocillopora damicornis* TspmSyn [XP_027059173]; OfSpmSyn, *Orbicella faveolata* SpmSyn [XP_027044059]; PdSpmSyn, *Pocillopora damicornis* SpmSyn [XP_027044059]; OfTspmSyn, *Orbicella faveolata* TspmSyn [XP_020625774]; HsSpdSyn, *Homo sapiens* SpdSyn [NP_003123]; PdSpdSyn, *Pocillopora damicornis* SpdSyn [XP_027050858]; SrSpdSyn, *Salpingoeca rosetta* SpdSyn [XP_004993732].

```
                       *        20         *        40         *        60         *        8
HvSpdSyn    : -------MEAETAAKRARESDGAA------AAADGAVEQAG-------ISAVIPGWFSEIS---------------PMW :  44
HsSpdSyn    : ------------------------------AAAGAGEQAG-------ISAVIPGWFSEIS---------------PMW :  26
AtSpdSyn1   : ---MDAKETSATDLKRPREEDDNG------GAATMETENGDQKKEPACFSTVIPGWFSEMS---------------PMW :  55
HvSpmSynX1  : MEGGDVRNGLTGTSQTKGSGVDSP--AKPLPPCCVKARAAAPESEAKCHATVVSGWFTETHSRCGKAS-KLQYYNNPMW :  76
HsSpmSyn    : MEGGDARNGLIGTAQIKGSGDDGS--TKPLPPCCVKARAAAPESEAKCHATVVSGWFTEPRSRCGKTS-KVQYYNNPMW :  76
HvSpmSyn2   : MEGGGARNVSAAAVQTKGTGDDGS--RKPLPPCCVKAQAAVAESEAKCHATVVSGWFTGTRSRSGKPS-KAQYFNNPMW :  76
AtSpmSyn    : MEG-DVGIGLVCQNTMDGKASNGNGLEKTVPSCCLKAMACVPEDDAKCHSTVVSGWFSEPHPRSGKKGGKAVYFNNPMW :  78
AtTspmSyn   : -------MGEAEVEMFG-------------NGFPEILKATSPTQTLHSNQQDCHWYEETID----------------- :  41

              0         *       100         *       120         *       140         *       1
HvSpdSyn    : PGEAHSLKVEKVLFQGKSDYQDVLVFQSSTYGKVLVLDGVIQVTERDECAYQEMITHLPLCSIKDPKKVLVIGGGDGGV : 123
HsSpdSyn    : PGEAHSLKVEKVLFQGKSDYQNVMVFQSSTYGKVLVLDGVIQVTERDECAYQEMITHLPLCSIKDPKKVLVIGGGDGGV : 105
AtSpdSyn1   : PGEAHSLKVEKVLFQGKSDYQDVIVFQSATYGKVLVLDGVIQLTERDECAYQEMITHLPLCSIPNPKKVLVIGGGDGGV : 134
HvSpmSynX1  : PGETHSLKVEKILYQGKSPYQEVLIFESLTYGKVLVLDGIVQLTDKDECAYQEMITHLPLCSIPSPKKVLVIGGGDGGV : 155
HsSpmSyn    : PGEAHSLKVENILYQGKSPYQEVLIFESSTYGKVLVLDGIVQLTDKDECAYQEMITHLPLCSIPSPKKVLVIGGGDGGV : 155
HvSpmSyn2   : PGEAHSLKVEKILYQGKSPYQEVLVFESSTYGNVLVLDGIVQLTDKDECAYQEMVTHLALCSIPSPKNVLVVGGGDGGV : 155
AtSpmSyn    : PGEAHSLKVEKVLFKDKSDFQEVLVFESATYGKVLVLDGIVQLTEKDECAYQEMIAHLPLCSISSPKNVLVVGGGDGGV : 157
AtTspmSyn   : DDLKWSFALNSVLHQGTSEYQDIALLDTKRFGKVLVIDGKMQSAERDEFIYHECLIHPALLFHPNPKTVFIMGGGEGSA : 120

              60        *       180         *       200         *       220         *
HvSpdSyn    : LREVSRHSSVEQIDICEIDKMVVDVSKQFFPHLALGFEDPRVSLHIGDGVAFLKNAPEGTYDAVIVDSSDPVG--PAQE : 200
HsSpdSyn    : LREVSRHSSVEQIDICEIDKMVVDVSKQFFPHLAVGFEDPRVSLHIGDGVSFLKNVPEGTYDAVIVDSSDPIG--PAQE : 182
AtSpdSyn1   : LREVARHASIEQIDMCEIDKMVVDVSKQFFPDVAIGYEDPRVNLVIGDGVAFLKNAAEGSYDAVIVDSSDPIG--PAKE : 211
HvSpmSynX1  : LREIARHGSVESIDICEIDQLVIDVCKDFFPDLSVGFKDPRVRLHVGDAVEFLRNTPEGTYDAIIVDSSDPIG--PAQE : 232
HsSpmSyn    : LREISRHGSVEFIDICEIDQL---VCKDFFPDLSVGFKDPRVQLHVGDAVEFLRNAPEGTYDAIIVDSSDPIG--PAQE : 229
HvSpmSyn2   : LREIAKHDSVESIDICEIDQLVIDVCKDFFPRLYVGYKDPRVRLHVGDAVEFLRNSPEGKYDAIIVDSSDPIG--PAQE : 232
AtSpmSyn    : LREISRHSSVEVIDICEIDKMVIDVSKKFFPELAVGFDDPRVQLHIGDAAEFLRKSPEGKYDAIIVDSSDPVG--PALA : 234
AtTspmSyn   : AREILKHTTIEKVVMCDIDQEVVDFCRRFLTVNSDAFCNKKLELVIKDAKAELEKREE-KFDIIVGDLADPVEGGPCYQ : 198

              240       *       260         *       280         *       300         *
HvSpdSyn    : LFEKPFFQSVSR-ALRPGGVVCTQA--ESIWLHMHIIEDIVTNCRQVFKGSVNYAWTTVPTYPSGVIGFMLCSTEGPSV : 276
HsSpdSyn    : LFEKPFFQSVAR-ALRPGGVVCTQA--ESIWLHMHIIEDIVANCRHVFKGSVNYAWTTVPTYPSGVIGFMLCSTEGPTV : 258
AtSpdSyn1   : LFEKPFFQSVAR-ALRPGGVVCTQA--ESLWLHMDIIEDIVSNCREIFKGSVNYAWTSVPTYPSGVIGFMLCSTEGPDV : 287
HvSpmSynX1  : LVEKPFFETIAR-ALRPGGVLCNQA--ESMWLHTHLIQDMLSICCETFKGSVHYAWTSVPTYPSGAIGFLLCAKEGPPV : 308
HsSpmSyn    : LVEKPFFETICR-ALRPGGVLCNQA--ESMWLHTHLIQDMLSICRETFKGSVHYAWTSVPTYPSGVIGFLLCAKEGPPV : 305
HvSpmSyn2   : LVEKPFFQAIAR-ALKPGGVLSNLA--ESMWLHTHLIQDMLSICREVFKGGVHYAWASVPTYPSGVIGFLLCAKEGPPV : 308
AtSpmSyn    : LVEKPFFETLAR-ALKPGGVLCNMA--ESMWLHTHLIEDMISICRQTFK-SVHYAWSSVPTYPSGVIGFVLCSTEGPAV : 309
AtTspmSyn   : LYTKSFYQNILKPKLSPNGIFVTQAGPAGIFTHKEVFTSIYNTMKQVFKYVKAYT-AHVPSFAD-TWGWVMASDHEFDV : 275

               320        *       340         *       360         *       380         *
HvSpdSyn    : DFQHPVFSIED-DEYSTKSKGPLKFYNSEFHTASFCLPSFARRVIEAKAN---------------------------- : 325
HsSpdSyn    : DFQHPVSNFEE-DEYSTKSKGLLKFYNSEIHSASFCLPSFAKRVIESKAN---------------------------- : 307
AtSpdSyn1   : DFKHPLNPID---ESSSKSNGPLKFYNAEIHSAAFCLPSFAKKVIESKAN---------------------------- : 334
HvSpmSynX1  : NFLAPINPIEK-LEGAMTAGRDIRFYNTEMHKAAFVLPTFAKRELETYGGSTRRAQQEETSAEPAKVAIVPHSEILTA : 385
HsSpmSyn    : NFLSPVNPIEK-LEGAMKAGREIRFYNSE------------------------------------------------- : 333
HvSpmSyn2   : DFLTPVNPIEK-IEGATKDGREMRFYNSEIHRAAFILPTFVKRELEAYNTSTEKEKLEKPTAKPVKMKVMRDSAITAS : 385
AtSpmSyn    : DFKNPINPIEK-LDGAMTHKRELKFYNSDMHRAAFALPTFLRREVASLLAS--------------------------- : 359
AtTspmSyn   : EVDEMDRRIEERVNGELMYLNAPSFVSAATLNKTISLALEKETEVYSEENARFIHGHGVAYRHI-------------- : 339
```

Figure S2. Amino acid sequence alignment of aminopropyltransferases from barley (*Hordeum vulgare*), wild barely (*H. spontaneum*) and *Arabidopsis thaliana*. Hv, *Hordeum vulgare* (barley); *Hs, Hordeum spontaneum* (wild barley); *At, Arabidopsis thaliana*. Genbank protein accession numbers: HvSpdSyn, XP_044960669; AtSpdSyn, NP_173794; HvSpmSynX1, XP_044945710; HvSpmSyn2, XP_044958835; AtSpmSyn, NP_568785; AtTspmSyn, OAO96167. The wild barley proteins were identified using BLASTP of the wild barely protein database: (http://www.ncgr.ac.cn/wild_barley) HsSpdSyn, protein model PH01000269G0650; HsSpmSyn, PH01003530G0160.

```
TspmSyn1     10 LSMHNKWFIERCDENFFAVSSLTGLEYTGTTKFQKMDVVTLGSFGRCLVLDDETQSSELD
TspmSyn2     10 LSRHNFFYYEYVMEGFRGESKLLSIDFSGKTKFQNMEILELESFGKCLVLDGEIQSAEKD
                 ** **    *   * *   * *      * **** *    * *** ***** * ** * *

TspmSyn1     70 EMIYHESLVHPAMLMATNPERVLIAGGGEGATLREVLRHKSVKEAVMVDIDGECVEQCKK
TspmSyn2     70 EHIYHESLVQPAMLSHPNPKRVFIAGGGEGATAREVIKHPSVEECVMVDIDGDCVEYSKK
                 * ******* ****   ** ** ********* ***  * ** * ******* ***  **

TspmSyn1    130 HLPQHHAGAFDDPRTTLIIDDANKIIQE-SDNESFDVIILDLADPVEGNPCFQLYAKSFY
TspmSyn2    130 LLKDHHKGALEHEKVKVIIDDAKHYLEDIAEDNSFDVMILDLADPVKGGPCFQLYSDSWY
                  *  ** **        *****           **** ******** * ******  * *

TspmSyn1    189 EMCKQKLRKGGVLVTQSGPAGLVTFRNVFTPIHNTLKQVFKRVSPYSTYVPSFIDNYGFT
TspmSyn2    190 ELCKRKLTKNGVLVTQSGCAGILTHDAILSPVHNTLKQVFSKVYPYSATIPSFCDDWGFN
                 * ** ** * ******** **  *      * ********  * ***   *** *  **

TspmSyn1    249 VVLKEDDSNLPDLTAVDPQWIDERINESISDPSI-LKHYDGISHRRMFNLPKQIRDGLSD
TspmSyn2    250 IAFQSEE----DAKALNDGQLDEKIEKRIEGGQTNMKFYDGISHTLLFALAKQIRQTIAK
                            *  *      ** *   *       * ******   * * ****

TspmSyn1    308 EKRVISKDNFIFM
TspmSyn2    306 EERVISKDNYVYV
```

Figure S3. Amino acid sequence alignment of thermospermine synthase paralogs from the excavate flagellate *Percolomonas cosmopolites*. The complete amino acid sequences are provided in Table S2.
